# Longitudinal Multi-Omics Profiling of Aqueous Humor Implicates GALNS Depletion as a Pro-Fibrotic Mediator of Anti-VEGF Therapy in PDR

**DOI:** 10.1167/iovs.67.5.43

**Published:** 2026-05-18

**Authors:** Yujuan Huang, Mi Gui, Xiangbin Kong, Taozheng Li, Zikang Xu, Jing Li, Bikun Xian, Yifan Zhang, Xuenan Zhuang, Liang Zhang, Dan Cao

**Affiliations:** 1 Guangdong Eye Institute, Department of Ophthalmology, Guangdong Provincial People’s Hospital (Guangdong Academy of Medical Sciences), Southern Medical University, Guangzhou, People's Republic of China; 2 Department of Ophthalmology, The Second People's Hospital of Foshan, Affiliated Foshan Hospital of Guangdong Pharmaceutical University, Foshan, Guangdong Province, People's Republic of China

**Keywords:** proliferative diabetic retinopathy (PDR), anti-VEGF therapy, crunch syndrome, multi-omics, *GALNS*

## Abstract

**Purpose:**

Preoperative anti-vascular endothelial growth factor (VEGF) therapy in proliferative diabetic retinopathy (PDR) may exacerbate fibrovascular contraction, posing significant challenges. This study aims to characterize proteo-metabolomic changes induced by anti-VEGF therapy, validate key candidates on orthogonal analytical platforms, and provide functional evidence for the lead molecule.

**Methods:**

Aqueous humor from 25 patients with PDR with simple vitreous hemorrhage (VH; *n* = 13) or VH with tractional retinal detachment (VH + TRD; *n* = 12) underwent data-independent acquisition (DIA) proteomics and widely targeted metabolomics before and 7 days after intravitreal aflibercept. Molecules altered in both cross-sectional and longitudinal comparisons were classified as “aggravation-type” or “improvement-type.” Aggravation-type molecules were validated by parallel reaction monitoring/selected reaction monitoring (PRM/SRM) in an independent cohort (cataract controls, *n* = 10; simple VH, *n* = 5; VH + TRD, *n* = 5), and *GALNS* was functionally assessed by siRNA knockdown in fibroblasts.

**Results:**

Intersection analysis identified 38 molecules (5 proteins and 33 metabolites): 35 improvement-type and 3 aggravation-type (CAST, GALNS, and 3-hydroxypropanoic acid [3-HPA]). Independent validation using PRM proteomics and SRM metabolomics confirmed that only *GALNS* was robustly validated across both cross-sectional (*P* = 0.016) and longitudinal (*P* = 0.049) comparisons, whereas CAST and 3-HPA did not reach statistical significance in validation. *GALNS* exhibited a biphasic pattern—elevated in PDR relative to cataract controls yet depleted in VH + TRD and further declined post-treatment. *GALNS* knockdown in fibroblasts upregulated α-SMA and collagen I, accelerated migration, and enhanced contractility.

**Conclusions:**

This multi-omics study, reinforced by independent validation, reveals *GALNS* depletion as a potential pro-fibrotic molecule following anti-VEGF therapy in PDR, offering a promising candidate for perioperative risk stratification. CAST and 3-HPA remain exploratory candidates requiring validation in larger cohorts.

Proliferative diabetic retinopathy (PDR) is a leading cause of severe vision impairment among working-age adults, marked by abnormal retinal neovascularization and progressive fibrovascular proliferation.^1^ In advanced cases, complications such as vitreous hemorrhage (VH) and tractional retinal detachment (TRD) often occur, for which pars plana vitrectomy (PPV) remains the standard surgical intervention.

To optimize surgical outcomes, intravitreal administration of anti-vascular endothelial growth factor (VEGF) agents before surgery has been widely adopted. Numerous studies have demonstrated its benefits in reducing intraoperative bleeding, shortening operative time, and lowering the rate of iatrogenic complications.^2^^,^^3^ Nevertheless, this therapy presents a clinical dilemma: in some patients, rapid fibrovascular membrane contraction follows injection, a phenomenon referred to as the “crunch syndrome.”^4^^,^^5^ This paradoxical response increases tractional forces and may worsen prognosis, yet the mechanisms driving this profibrotic shift are still poorly understood.

Most molecular studies on PDR have emphasized the angiogenic pathway or relied on single-modality analyses, which cannot fully capture the intricate biological interactions that shape disease progression.^6^^,^^7^ These approaches often overlook the heterogeneity between patients with distinct baseline phenotypes (e.g., VH versus TRD) and seldom incorporate longitudinal follow-up, making it difficult to determine whether adverse events reflect pre-existing risk or treatment-induced alterations. Furthermore, the reliance on isolated datasets limits the ability to dissect the interplay among angiogenesis, extracellular matrix (ECM) remodeling, and metabolic changes.^8^^,^^9^ Moreover, candidate biomarkers emerging from discovery-phase omics studies have rarely been independently validated on orthogonal analytical platforms or substantiated by functional experiments, constraining their translational utility.

Multi-omics profiling of ocular biofluids offers a promising strategy to bridge these gaps.^10^ Aqueous humor, in particular, provides a dynamic window into the vitreo-retinal microenvironment and allows simultaneous evaluation of proteins and metabolites that reflect treatment response.^11^^,^^12^ By integrating proteomic and metabolomic data, it becomes possible to separate baseline molecular signatures from therapy-induced changes and to classify responses according to their clinical impact.

Accordingly, this study was designed with five objectives: (1) to characterize baseline molecular heterogeneity across severe PDR phenotypes, (2) to map longitudinal proteo-metabolomic changes following anti-VEGF therapy, (3) to identify pathways associated with either clinical improvement or worsening fibrosis, (4) to independently validate key candidate molecules using targeted analytical platforms in an expanded cohort that includes non-diabetic controls, and (5) to provide preliminary functional evidence for the pro-fibrotic role of the most robustly validated candidate. We propose that, whereas anti-VEGF treatment exerts predominantly beneficial effects, it may concurrently trigger specific pro-fibrotic programs that explain the occurrence of crunch syndrome. This framework aims to advance the molecular basis for personalized perioperative management of PDR.

## Methods

### Study Design and Participants

This prospective, dual-center study utilized a baseline-stratified, longitudinal paired design, enrolling consecutive patients from May 2024 to November 2025 at Guangdong Provincial People’s Hospital and the Second People’s Hospital of Foshan. The discovery cohort comprised patients with PDR complicated by VH, who were scheduled for a preoperative anti-VEGF injection followed by a planned PPV 7 days later. An independent validation cohort was subsequently enrolled, comprising non-diabetic patients with cataract undergoing phacoemulsification and patients with PDR. The study protocol was approved by the institutional review boards of Guangdong Provincial People’s Hospital (Approval No. KY2024-797-02) and the Second People’s Hospital of Foshan (Approval No. (2024)-0052), and all participants provided written informed consent.

Inclusion criteria were PDR with persistent VH requiring PPV and an age between 20 and 79 years. Exclusion criteria included: (1) any history of intravitreal injection or retinal photocoagulation; (2) a history of vitrectomy; (3) HbA1c levels exceeding 12% in patients with diabetic retinopathy; (4) concurrent serious ocular diseases other than PDR, including glaucoma, macular degeneration, or other retinal vascular diseases; (5) presence of ocular infection or intraocular inflammation; and (6) severe uncontrolled systemic diseases, including uncontrolled diabetes, uncontrolled hypertension, or myocardial infarction or cerebrovascular accident within the past 6 months. Preoperative classification into simple VH and VH + TRD was based on comprehensive ophthalmic assessment including indirect ophthalmoscopy and, when fundus visualization was precluded by dense vitreous hemorrhage, B-scan ultrasonography (Quantel Medical, Aviso S). The final diagnosis was confirmed intraoperatively during PPV.

### Anti-VEGF Treatment and Aqueous Humor Sampling

Paired aqueous humor samples (120–150 µL) were prospectively collected from each participant at two key time points: immediately before the anti-VEGF injection (baseline) and 7 days later at the start of the PPV procedure (post-treatment). All participants received a single intravitreal injection of aflibercept (2 mg/0.05 mL) administered under sterile conditions, 3.5 to 4.0 mm posterior to the corneal limbus, preferentially in the superotemporal quadrant. Both baseline and post-treatment samples were aspirated slowly using a 30-gauge insulin syringe. Any sample contaminated with blood was immediately discarded. Following collection, samples were placed on ice and transferred for storage at −80°C within 15 to 30 minutes. Each sample was assigned a unique identifier and tracked using a dedicated chain-of-custody form to ensure sample integrity.

### Data-Independent Acquisition-Based Proteomics Workflow

Aqueous humor proteins were extracted, reduced with DTT, alkylated with IAA, and digested overnight with trypsin. Peptides were analyzed on a Vanquish Neo UHPLC system coupled to an Orbitrap Astral mass spectrometer (Thermo Fisher Scientific) operated in data-independent acquisition (DIA) mode with 299 isolation windows (2 Th width). Protein identification and quantification were performed using DIA-NN (version 1.8.1) in library-free mode against the UniProt human proteome database (UP000005640; 83,386 entries), with false discovery rate (FDR) controlled at <1% at both precursor and protein levels. A total of 9440 unique peptides and 1432 protein groups were identified and quantified. All samples were analyzed in a single analytical batch with randomized injection order and pooled quality control (QC) injections; instrument stability was confirmed by iRT peptide monitoring (overall CV = 2.6%; Supplementary Fig. S2). Full sample preparation, liquid-chromatography mass spectrometry (LC-MS) acquisition parameters, data processing, and QC metrics are provided in the Supplementary Methods.

### Widely Targeted Metabolomics

Aqueous humor metabolites were extracted using acetonitrile:methanol (1:4, v/v) containing isotope-labeled internal standards. Widely targeted metabolomic profiling was performed using an integrated ultraperformance liquid chromatography-tandem mass spectrometry (UPLC-MS/MS) approach, as previously described.^13^^–^^15^ Briefly, metabolite annotation was first performed on a TripleTOF 6600 system (AB SCIEX) in IDA mode against the MetWare in-house database (MedDB version 7.5), and annotated MRM ion pairs were then monitored on a QTRAP 6500+ system (SCIEX) for scheduled MRM quantification. A total of 1542 metabolites were quantified. Metabolite identification confidence was classified according to a 4-tier system aligned with MSI criteria:16; 58.4% (*n* = 901) were identified at MSI level 1 against authenticated reference standards, and 41.6% (*n* = 641) were putatively annotated at MSI level 2 to 3. QC samples demonstrated excellent reproducibility (Pearson *r* = 0.977–0.998, >85% peaks with CV < 0.3; Supplementary Fig. S3). Full acquisition parameters, chromatographic conditions, and QC metrics are provided in the Supplementary Methods.

For both proteomic and metabolomic datasets, data were processed independently. Features present in over 50% of samples within at least one group were retained; and the remaining features were removed to eliminate noise from sporadic, low-confidence identifications. Missing values were imputed using the K-nearest neighbors (KNN) algorithm. Protein abundance data were log2-transformed and median-normalized; metabolite data were log2-transformed and quantile-normalized.

### PRM-Based Targeted Proteomics Validation

To independently validate the aggravation-type proteins CAST and GALNS identified from DIA discovery, parallel reaction monitoring (PRM) was performed on an independent analytical platform. Aqueous humor proteins were processed using SP3 (Single-Pot Solid-Phase-enhanced Sample Preparation), and peptides were separated on a Vanquish Neo nano-UPLC system and analyzed on a Q Exactive HF-X mass spectrometer (Thermo Fisher Scientific) in PRM mode. Three proteotypic peptides per protein were monitored; all achieved a 100% detection rate across all 30 runs. Instrument stability was confirmed by iRT peptide monitoring (overall CV = 2.6%; Supplementary Fig. S4). Full PRM method development, acquisition parameters, and data analysis details are provided in the Supplementary Methods.

### Targeted Metabolite Quantification

To independently validate 3-hydroxypropanoic acid (3-HPA), targeted absolute quantification was performed using UPLC-MS/MS with selected reaction monitoring (SRM) on the AccuQuanter platform (iPhenome Biotechnology Inc., Dalian, China). Samples underwent chemical derivatization with 3-nitrophenylhydrazine (3-NPH), and analysis was performed on a Thermo Ultimate 3000 UPLC coupled to a TSQ Endura MD Plus triple quadrupole MS (Thermo Fisher Scientific). Isotope-labeled internal standards (3-HPA-D4 sodium salt) and multi-point calibration curves were used for absolute quantification (nmol/L). QC samples demonstrated excellent precision (CV < 20%; Supplementary Fig. S5). Full sample preparation, derivatization, chromatographic, and MS parameters are provided in the Supplementary Methods.

### In Vitro Functional Validation of GALNS Depletion

To investigate the functional consequence of GALNS reduction on fibrotic behavior, L929 murine fibroblasts (Cellcook, Guangzhou, China) were cultured in high-glucose DMEM (Gibco) supplemented with 10% horse serum and 1% penicillin/streptomycin at 37°C in a humidified atmosphere containing 5% CO₂. GALNS expression was silenced by transfecting cells with a specific siRNA targeting mouse Galns (si-GALNS; sense: 5′-CCUUUGUGCUCACCAUCUAtt-3′) or a scrambled negative control siRNA (si-NC), both synthesized by PackGene Biotech (Guangzhou, China), at a final concentration of 50 nM using CALNP RNAi Transfection Reagent (Beijing Nuona Pharmaceutical Technology) per the manufacturer’s protocol. Knockdown efficiency and downstream fibrotic marker expression—including α-smooth muscle actin (α-SMA) and collagen I—were assessed 48 hours post-transfection by Western blotting, performed as previously described with anti-GALNS (1:1000, Abcam, ab231647), anti-α-SMA (1:2000, Proteintech, 14395-1-AP), anti-collagen I (1:2000, ABclonal, A1352), and anti-GAPDH (1:10000, Proteintech, 60004-1-I*g*) antibodies. Cell migratory capacity was evaluated by a scratch wound healing assay: confluent monolayers were scratched with a sterile 200-µL pipette tip 48 hours post-transfection, washed with PBS, and maintained in DMEM containing 1% horse serum. Wound images were captured at 0, 24, and 48 hours using an inverted microscope (Nikon), and wound closure rate was calculated as [(Area_0h_ − Area_t_)/Area_0h_] × 100%. Cell contractility was further assessed using a collagen gel contraction assay (CBA-201; Cell Biolabs, San Diego, CA, USA) following the manufacturer’s instructions; transfected cells were embedded in collagen lattices under stressed conditions for 48 hours prior to gel release, and the relative gel area was quantified at 0, 24, and 48 hours post-release using ImageJ software.

### Statistical Analysis and Visualization

Statistical analyses addressed two primary comparisons: a cross-sectional comparison between VH + TRD and simple VH groups at baseline, and a longitudinal comparison between post-anti-VEGF and baseline samples. Appropriate unpaired and paired statistical tests were applied, respectively, after assessing data for normality and homogeneity of variance. Differential molecules were identified using a dual-threshold approach. For proteomics, features were considered significant with a *P* < 0.05 and a fold change (FC) >1.5. For metabolomics, significance was defined by a *P* < 0.05 combined with a variable importance in projection (VIP) score >1 from our PLS-DA model. Molecules meeting the significance criteria in both comparisons were identified through intersection analysis and classified as “improvement-type” or “aggravation-type” based on their directional changes relative to disease pathology. Pathway enrichment analysis was conducted for proteins, metabolites, and their combination using the Kyoto Encyclopedia of Genes and Genomes (KEGG) Pathway database via an over-representation analysis (*P* < 0.05). All data visualization and analyses were performed in R software, utilizing packages such as ggplot2, cowplot, ggrepel, ggforce, and ggpattern. Multivariable sensitivity analyses (ANCOVA adjusting for age, sex, diabetes duration, and HbA1c) were performed for cross-sectional comparisons. Post hoc power analysis, Cohen’s d effect sizes with 95% confidence intervals, and prospective sample size estimation were calculated for all key comparisons using the R pwr package (2-sided α = 0.05, 80% power target); details are provided in Supplementary Figure S6.

## Results

### Patient Characteristics

As shown in the Table, the discovery cohort included 25 patients with PDR patients (simple VH, *n* = 13 and VH + TRD, *n* = 12). The two groups were comparable in age (54.7 ± 6.8 vs. 51.9 ± 7.0 years, *P* = 0.252), sex, diabetes duration (9.1 ± 4.2 vs. 6.8 ± 5.8 years, *P* = 0.106), HbA1c (8.6 ± 2.0% vs. 7.8 ± 1.9%, *P* = 0.481), body mass index (BMI), blood pressure, and other clinical parameters (all *P* > 0.05; see the Table). All patients with PDR were treatment-naive. The independent validation cohort (non-DR cataract controls, *n* = 10; simple VH, *n* = 5; and VH + TRD, *n* = 5) was similarly balanced across all available clinical variables (all *P* > 0.05; see the Table).

**Table.** Baseline Clinical Characteristics of Enrolled Patients

	Discovery Phase (DIA-Based Proteomics + Widely Targeted Metabolomics)			Validation Phase (PRM-Based Proteomics + Targeted Metabolomics)			
Variables	Simple VH (*n* = 13)	VH + TRD (*n* = 12)	*P* Value	Control (*n* = 10)	Simple VH (*n* = 5)	VH + TRD (*n* = 5)	*P* Value
Sample size, *n*	13	12	–	10	5	5	–
Age, y	54.7 ± 6.8	51.9 ± 7.0	0.252	51.1 ± 5.3	50.0 ± 3.2	51.0 ± 2.9	0.880
Sex, F *n* (%)	2 (15.4)	6 (50.0)	0.097	2 (20.0)	1 (20.0)	1 (20.0)	1.000
Eye (right), *n* (%)	9 (69.2)	8 (66.7)	1.000	6 (60.0)	3 (60.0)	3 (60.0)	1.000
Diabetes duration, y	9.1 ± 4.2	6.8 ± 5.8	0.106	–	9.3 ± 5.0	9.3 ± 9.5	1.000
Smoking, *n* (%)	0 (0.0)	2 (28.6)	0.220	3 (30.0)	1 (25.0)	3 (75.0)	0.493
Alcohol, *n* (%)	1 (25.0)	1 (14.3)	1.000	4 (40.0)	3 (75.0)	1 (25.0)	0.443
BMI, kg/m²	27.2 ± 9.2	23.7 ± 2.7	0.331	23.5 ± 2.3	24.0 ± 0.9	23.9 ± 1.1	0.886
Systolic BP, mm Hg	131.3 ± 13.1	138.2 ± 16.6	0.259	129.6 ± 11.8	134.5 ± 10.8	142.8 ± 20.4	0.289
Diastolic BP, mm Hg	77.3 ± 12.3	82.7 ± 6.7	0.203	76.5 ± 8.8	85.0 ± 8.5	84.0 ± 14.8	0.268
Insulin therapy, *n* (%)	0 (0.0)	2 (25.0)	0.220	–	0 (0.0)	0 (0.0)	1.000
Dialysis history, *n* (%)	1 (16.7)	0 (0.0)	1.000	0 (0.0)	0 (0.0)	0 (0.0)	1.000
HbA1c, %	8.6 ± 2.0	7.8 ± 1.9	0.481	–	8.4 ± 2.2	7.3 ± 1.7	0.548

DIA, data-independent acquisition (DIA-based proteomics); mm Hg, millimeters of mercury; PRM, parallel reaction monitoring (PRM-based proteomics); TRD, tractional retinal detachment; VH, vitreous hemorrhage; WT, widely targeted metabolomics.

Data are presented as mean ± SD for continuous variables, and *n* (%) for categorical variables.

Discovery phase P: Wilcoxon rank-sum test (continuous) or Fisher’s exact test (categorical) comparing simple VH versus VH + TRD.

Validation phase P: Kruskal-Wallis test (continuous) or Fisher’s exact test (categorical) across three groups.

Control group: patients with cataract without diabetes; diabetes-specific variables not applicable (“−”).

### Discovery-Phase Proteo-Metabolomic Profiling

**Figure 1.**
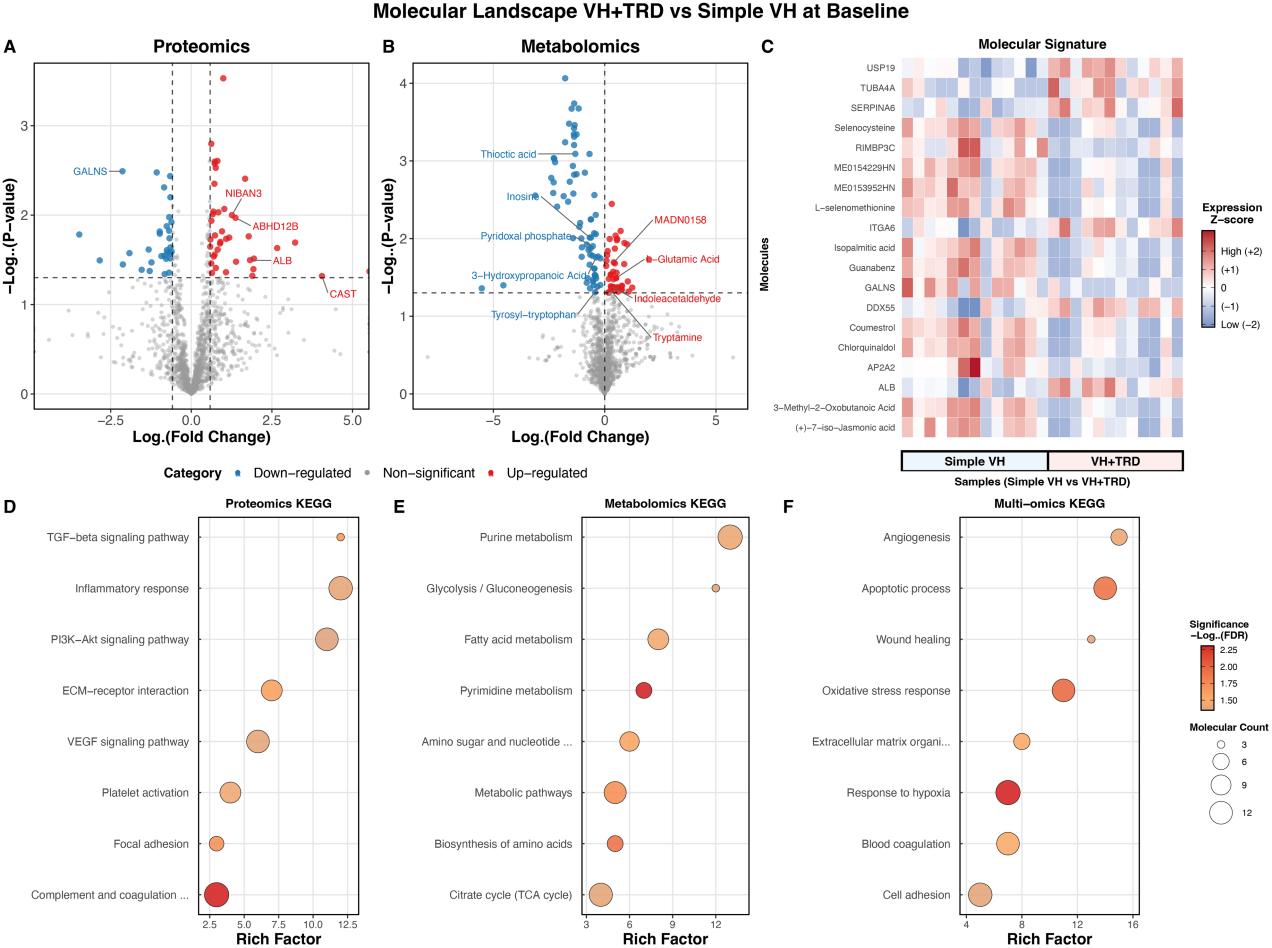
**Baseline differences in proliferative diabetic retinopathy: proteomic and metabolomic differences between VH** **+** **TRD and** **simple VH groups.** (**A, B**) Volcano plots highlighting significant differences in aqueous humor proteins and metabolites between VH + TRD and simple VH groups, respectively. *Red dots* indicate significantly upregulated molecules in the VH + TRD group (P *<* 0.05 and fold change > 1.5 for proteins, *P* < 0.05, and VIP > 1 for metabolites), *blue dots* represent significantly downregulated molecules, and *gray dots* denote nonsignificant changes. (**C**) Heatmap showing the top 20 molecules with the greatest differences among groups, including both proteins and metabolites. (**D–F**) KEGG pathway enrichment analysis of differential proteins, metabolites, and combined proteins and metabolites, respectively, showing the top enriched pathways ranked by −log10 (*P* value). VH + TRD, subretinal detachment with vitreous hemorrhage; KEGG, Kyoto Encyclopedia of Genes and Genomes; VIP, variable importance in projection.

At baseline, cross-sectional comparison between the VH + TRD and simple VH groups identified 71 differentially expressed proteins (DEPs) and 114 differentially expressed metabolites (DEMs; Figs. 1A, 1B; Supplementary Table S1). Among the DEPs, CAST was significantly upregulated in the VH + TRD group (*P* = 0.048), whereas GALNS was significantly downregulated (*P* = 0.003). A heatmap displays the top 20 molecules with the greatest baseline differences (Fig. 1C). KEGG pathway enrichment analysis of the baseline differential molecules revealed enrichment in ECM organization and TGF-β signaling for proteins, and metabolic pathways for metabolites (Figs. 1D–F; Supplementary Table S3).

**Figure 2.**
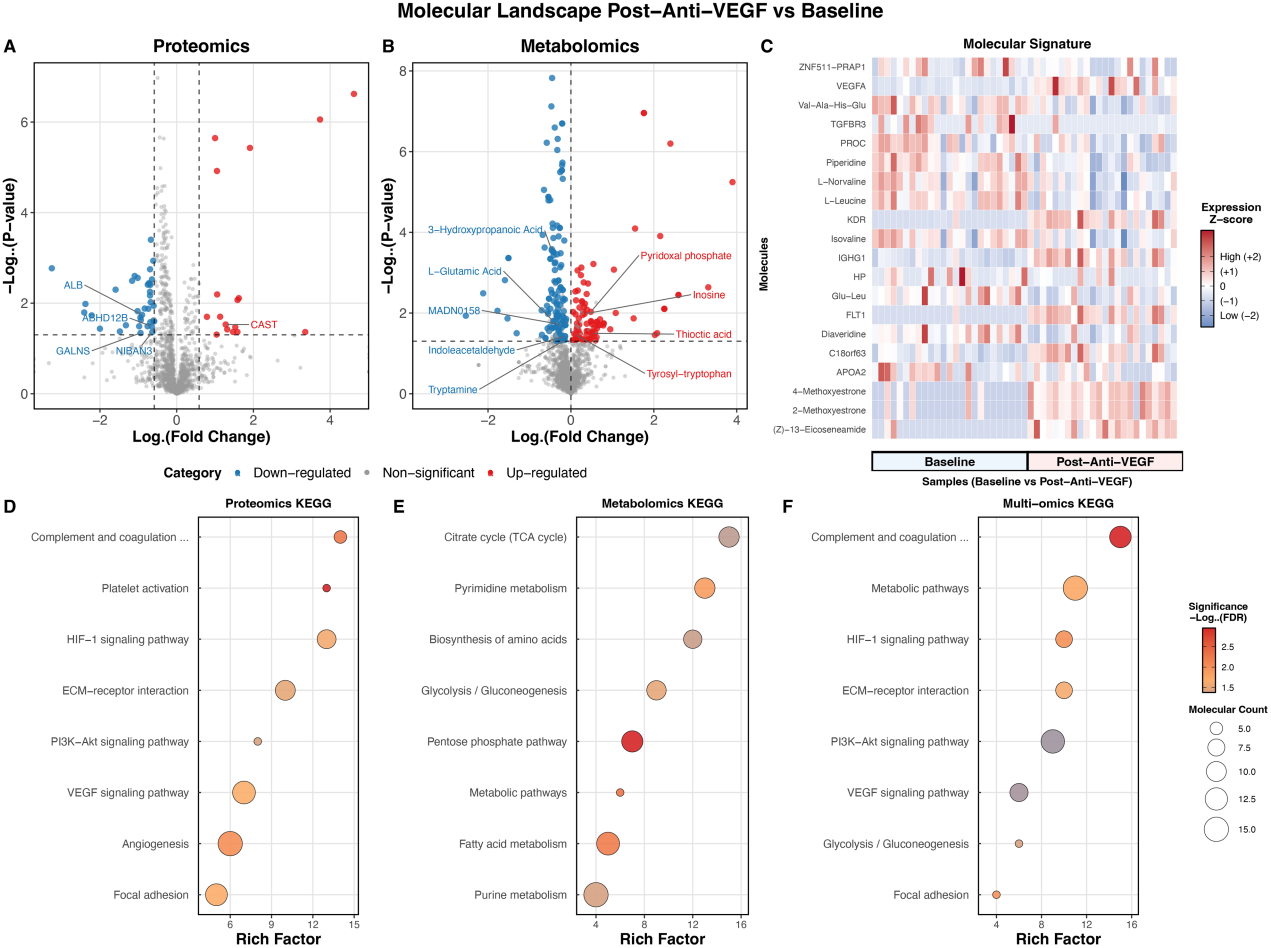
**Longitudinal molecular responses to anti-VEGF therapy in proliferative diabetic retinopathy.** (**A, B**) Volcano plots highlighting significant differences in aqueous humor proteins and metabolites between baseline and post-anti-VEGF treatment, respectively. *Red dots* indicate significantly upregulated molecules post-treatment (*P* < 0.05 and fold change > 1.5 for proteins, *P* < 0.05, and VIP > 1 for metabolites), *blue dots* represent significantly downregulated molecules, and *gray dots* denote nonsignificant changes. (**C**) Heatmap showing the top 20 molecules with the greatest differences between baseline and post-treatment, including both proteins and metabolites. (**D–F**) KEGG pathway enrichment analysis of differential proteins, metabolites, and combined proteins and metabolites, respectively, showing the top enriched pathways ranked by −log10 (*P* value). VEGF, vascular endothelial growth factor; KEGG, Kyoto Encyclopedia of Genes and Genomes; VIP, variable importance in projection.

Longitudinal analysis of all 25 paired aqueous humor samples identified 54 proteins and 220 metabolites significantly altered 7 days after anti-VEGF treatment (Figs. 2A, 2B; see Supplementary Table S1). The top 20 longitudinally altered molecules are shown in a heatmap (Fig. 2C). KEGG pathway analysis indicated significant changes in VEGF signaling, angiogenesis, and inflammatory response pathways (Figs. 2D–F; see Supplementary Table S3).

### Intersection Analysis and Molecular Classification

**Figure 3.**
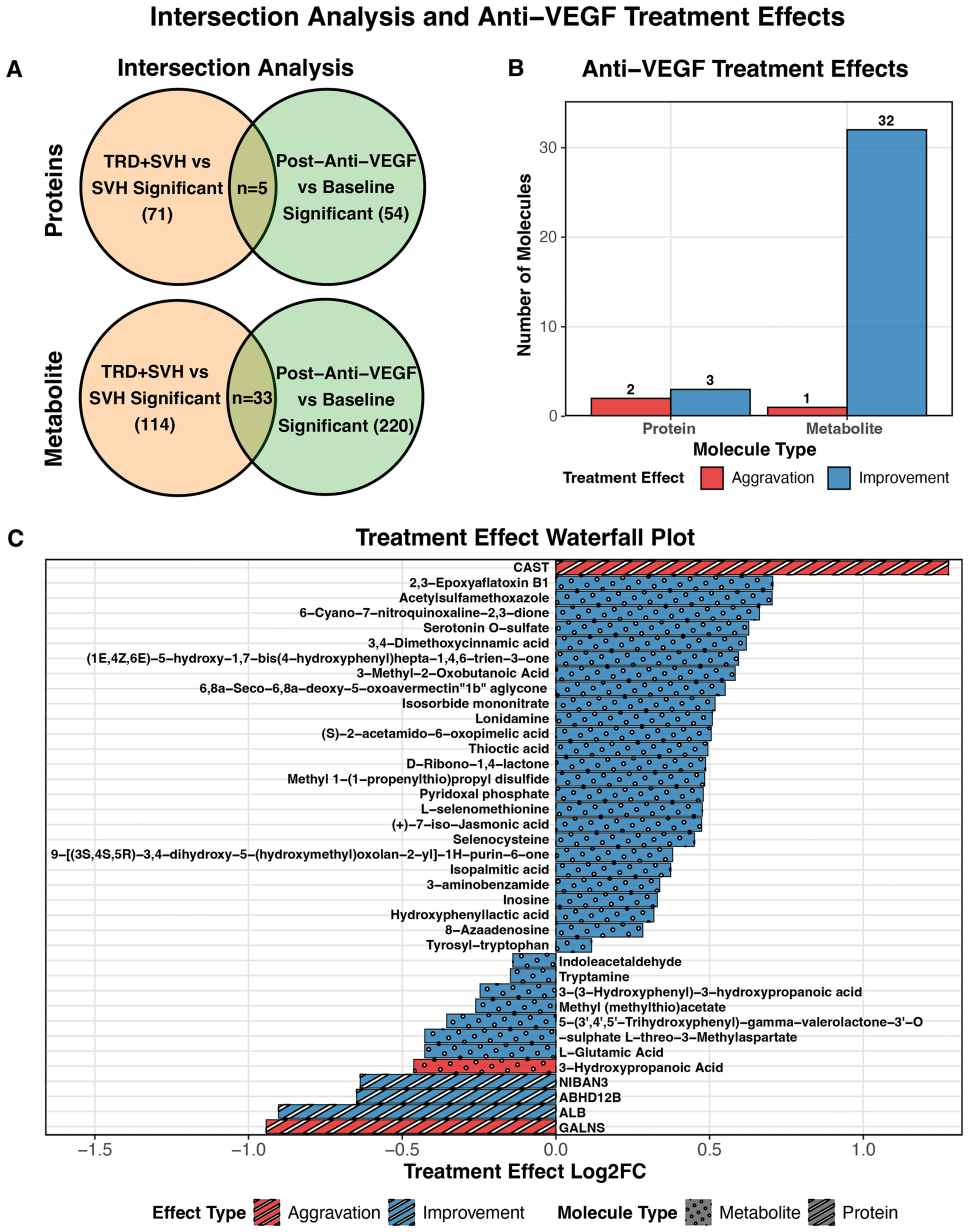
**Intersection analysis and effect-direction classification of molecules responding to anti-VEGF therapy.** (**A**) Venn diagrams showing the intersection of significantly altered proteins and metabolites between baseline comparison (VH + TRD versus simple VH) and longitudinal comparison (post-anti-VEGF versus baseline). Numbers indicate the count of molecules in each category, with the overlapping regions representing molecules significantly altered in both comparisons. (**B**) Bar chart displaying the classification of intersection molecules into improvement-type and aggravation-type based on their directional changes across both comparisons. Aggravation-type molecules (*n* = 3, including 2 proteins and 1 metabolite) and improvement-type molecules (*n* = 35, including 3 protein and 32 metabolites) show distinct directional changes across both comparisons. (**C**) Waterfall plot displaying the fold change patterns of all intersection molecules, ordered by their classification and magnitude of change. *Blue bars* represent improvement-type molecules and *red bars* represent aggravation-type molecules. *Striped pattern* indicates proteins whereas the *dotted pattern* indicates metabolites. VH + TRD, subretinal detachment with vitreous hemorrhage; VEGF, vascular endothelial growth factor.

Intersection analysis between the baseline differential molecules (VH + TRD versus simple VH) and the longitudinally altered molecules (post-treatment versus baseline) identified 5 proteins and 33 metabolites common to both comparison sets (Fig. 3A). Based on their directional changes, these 38 intersection molecules were classified into 2 categories: 35 “improvement-type” molecules, in which treatment reversed the pathological dysregulation observed in the VH + TRD group, and 3 “aggravation-type” molecules, in which treatment exacerbated baseline pathological patterns (Fig. 3B). A waterfall plot illustrates the FC patterns for all intersection molecules (Fig. 3C).

**Figure 4.**
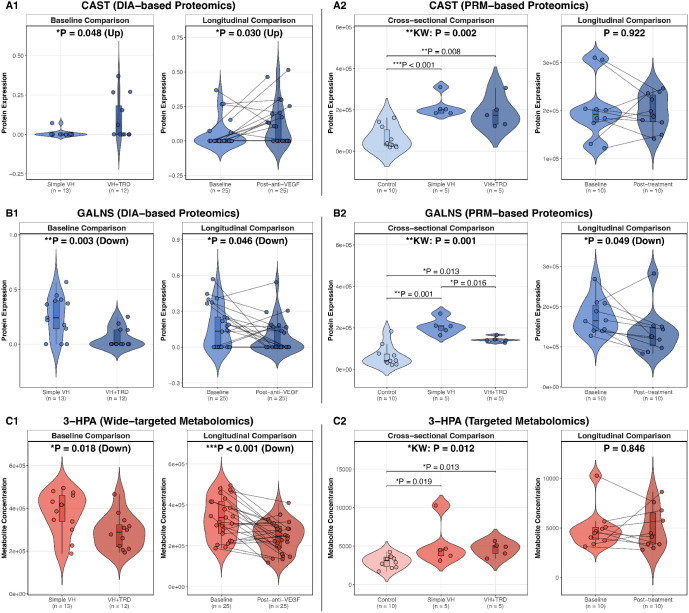
**Independent validation of three key aggravation-type molecules across discovery and targeted validation platforms.** Each row represents one molecule: (**A**) CAST, (**B**) GALNS, and (**C**) 3-hydroxypropanoic (3-HPA) acid. For each molecule, the left half (panels labeled 1) shows discovery-phase results from DIA-based proteomics (CAST and GALNS) or widely targeted metabolomics (3-HPA), and the right half (panels labeled 2) shows validation-phase results from PRM-based proteomics (CAST and GALNS) or targeted metabolomics (3-HPA). Within each half, the *left panel* displays cross-sectional comparisons and the *right panel* displays longitudinal paired comparisons. In the discovery phase, cross-sectional comparisons (simple VH versus VH + TRD; *n* = 13 and *n* = 12, respectively) were assessed using independent *t*-tests, and longitudinal comparisons (baseline versus post-anti-VEGF; *n* = 25 pairs) were assessed using paired *t*-tests. In the validation phase, cross-sectional comparisons among three groups (control, simple VH, and VH + TRD; *n* = 10, 5, and 5, respectively) were assessed using the Kruskal-Wallis test followed by pairwise Wilcoxon rank-sum tests; longitudinal comparisons (baseline versus post-treatment; *n* = 10 pairs) were assessed using paired Wilcoxon signed-rank tests. Data are presented as violin plots overlaid with boxplots and individual data points; box plots display the median (*horizontal line*), interquartile range (IQR; *box*), and whiskers extending to 1.5 × IQR. In longitudinal panels, lines connect paired observations from the same patient before and after anti-VEGF treatment. *Blue color scheme*: protein panels (CAST and GALNS); and *red color scheme*: metabolite panels (3-HPA). *Lighter shades* in the three-group validation panels indicate control samples. Significance levels: *P < 0.05, **P < 0.01, ***P < 0.001; KW, Kruskal-Wallis test. *P* values and trend directions (up or down) are annotated above each panel; significance *brackets* with *P* values are shown for pairwise comparisons in three-group panels when the overall Kruskal-Wallis test was significant (*P* < 0.05).

The three aggravation-type molecules exhibited consistent pathological trends across both comparisons. CAST expression was higher in the VH + TRD group at baseline (*P* = 0.048) and further increased after anti-VEGF treatment (*P* = 0.030; Fig. 4A1). GALNS expression was lower in the VH + TRD group at baseline (*P* = 0.003) and showed a continued decrease post-treatment (*P* = 0.046; Fig. 4B1). The 3-HPA acid levels were lower in the VH + TRD group at baseline (*P* = 0.018) and were further reduced after treatment (*P* < 0.001; Fig. 4C1). Among the 35 improvement-type molecules (Supplementary Fig. S1), representative examples include ALB, which was elevated in the VH + TRD group at baseline (*P* = 0.002) and decreased following treatment (*P* = 0.044); NIBAN3, which was upregulated at baseline (*P* = 0.010) and downregulated post-treatment (*P* = 0.044); and L-glutamic acid, which was elevated at baseline (*P* = 0.032) and decreased after treatment (*P* = 0.016). Complete statistical details for all 38 intersection molecules, including ANCOVA-adjusted *P* values controlling for age, sex, diabetes duration, and HbA1c, are provided in Supplementary Table S2.

### Independent Targeted Validation of Aggravation-Type Biomarkers

PRM-based targeted proteomics was performed for CAST and GALNS, and SRM-based targeted absolute quantification with isotope-labeled calibration curves was performed for 3-HPA acid in the independent validation cohort (Supplementary Table S4, see Fig. 4).

In the cross-sectional three-group comparison, Kruskal–Wallis tests revealed significant overall differences for all three molecules (CAST: *P* = 0.002; GALNS: *P* = 0.001; and 3-HPA: *P* = 0.012). Pairwise comparisons demonstrated that all three molecules were significantly elevated in both PDR subgroups relative to non-diabetic controls (all *P* < 0.05; Figs. 4A2, 4B2, 4C2). For the within-PDR subgroup comparison (VH + TRD versus simple VH), only GALNS achieved statistical significance (FC = 0.69, *P* = 0.016), concordant with the DIA discovery finding (*P* = 0.003; Figs. 4B1 vs. 4B2); CAST (*P* = 0.222), and 3-HPA (*P* = 0.690) showed nonsignificance.

In the longitudinal comparison (post-treatment versus baseline, 10 pairs), only GALNS showed a significant decrease after anti-VEGF treatment (FC = 0.78, *P* = 0.049), concordant with the DIA discovery (*P* = 0.046; Figs. 4B1 vs. 4B2). CAST (*P* = 0.922) and 3-HPA (*P* = 0.846) did not reach significance.

### GALNS Depletion Promotes Pro-Fibrotic Phenotype In Vitro

**Figure 5.**
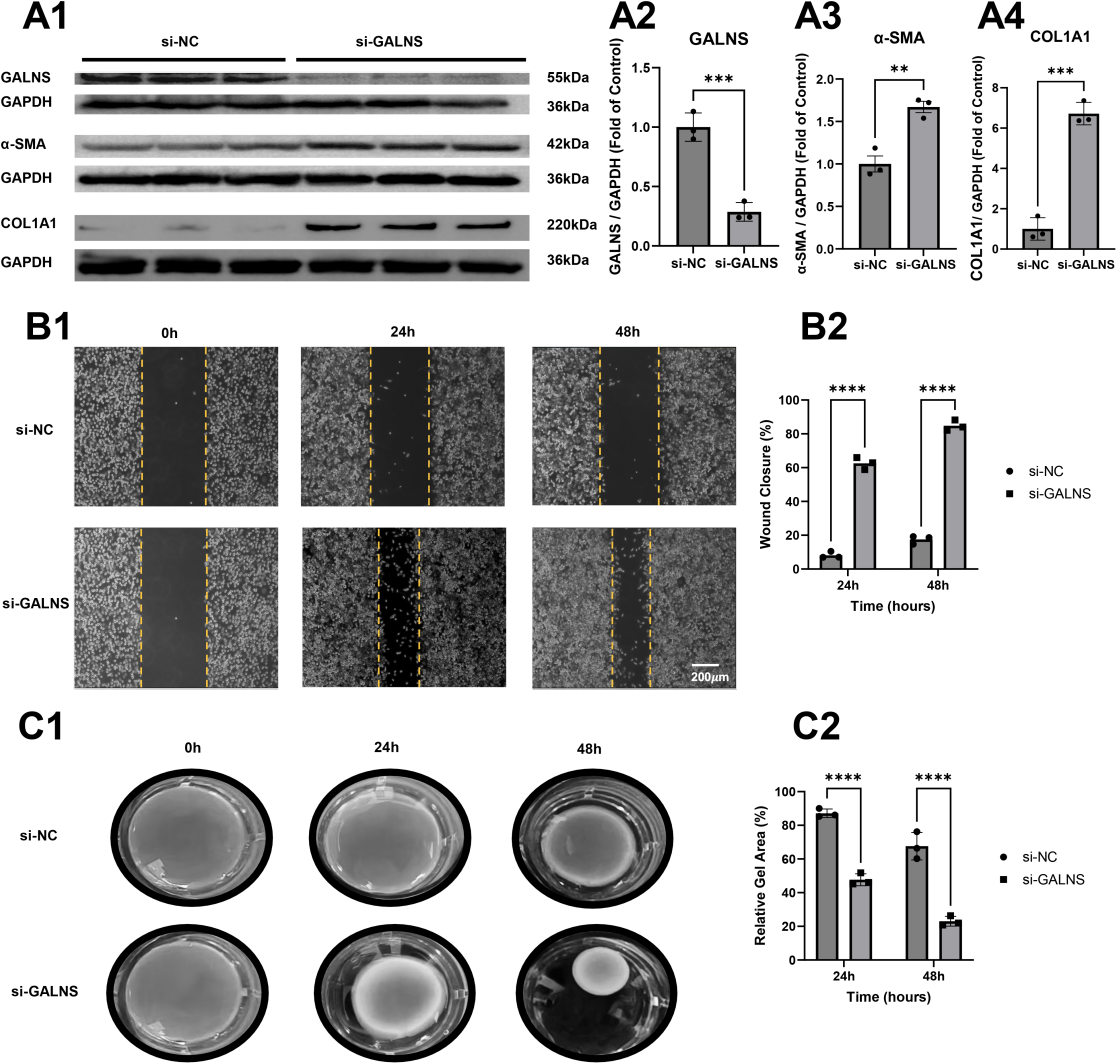
**GALNS knockdown promotes fibrotic activation, migration, and contractile phenotypes in L929 fibroblasts.** (**A**) Western blot analysis: (**A1**) representative blots for GALNS, α-SMA, COL1A1, and GAPDH; (**A2–A4**) quantification showing GALNS knockdown (***P < 0.001), α-SMA upregulation (**P < 0.01), and COL1A1 upregulation (***P < 0.001). (**B**) Scratch wound healing assay: (**B1**) Representative images at 0, 24, and 48 hours; *scale bar* = 200 µm; (**B2**) quantification of wound closure (****P < 0.0001). (**C**) Collagen gel contraction assay: (**C1**) macroscopic images at 0, 24, and 48 hours post-release; (**C2**) quantification of relative gel area (****P < 0.0001). Data: mean ± SD, *n* = 3; unpaired two-tailed Student’s *t*-test.

To functionally validate the aggravation-type role of GALNS, we performed siRNA-mediated knockdown in L929 murine fibroblasts (Fig. 5). Western blot analysis confirmed efficient GALNS knockdown (0.29 ± 0.08-fold of control, *P* < 0.001; see Figs. 5A1, 5A2). GALNS-depleted cells exhibited significant upregulation of α-SMA (1.67 ± 0.11 vs. 1.00 ± 0.15, *P* < 0.01; see Fig. 5A3) and collagen I (COL1A1; 6.72 ± 0.53 vs. 1.00 ± 0.55, *P* < 0.001; see Fig. 5A4).

In the scratch wound healing assay, GALNS knockdown markedly accelerated wound closure. At 24 hours, the wound closure rate in si-GALNS cells was 62.58 ± 3.54% compared to 8.16 ± 1.73% in si-NC controls (*P* < 0.0001), and by 48 hours these values reached 84.77 ± 3.02% and 17.53 ± 2.24%, respectively (*P* < 0.0001; see Figs. 5B1, 5B2).

In the collagen gel contraction assay, GALNS-depleted cells showed significantly greater gel contraction. The relative gel area at 24 hours post-release was 48.68 ± 2.83% for si-GALNS vs. 87.15 ± 2.38% for si-NC (*P* < 0.0001), decreasing further to 23.01 ± 2.78% vs. 67.59 ± 8.03% at 48 hours (*P* < 0.0001; see Figs. 5C1, 5C2).

## Discussion

This study used an integrated proteo-metabolomic approach to systematically dissect the molecular response to preoperative anti-VEGF therapy in PDR, utilizing a novel design that combines baseline stratification with longitudinal analysis. Our principal finding is the coexistence of a predominantly beneficial molecular signature with a smaller, but distinct, set of potentially detrimental signals. Of the 38 molecules intersecting both analyses, 92.1% were classified as “improvement-type,” aligning with the well-established clinical benefits of this therapy.^13^ However, a critical 7.9% of molecules were “aggravation-type,” implicating specific pro-fibrotic programs in the clinically observed crunch syndrome.^14^ Independent targeted validation on orthogonal platforms and in vitro functional experiments converged on GALNS as the most robust aggravation-type molecule, establishing it as a key pro-fibrotic mediator in this therapeutic context.

Among the three aggravation-type molecules, GALNS received the most robust and convergent support. The progressive downregulation of GALNS suggests impaired degradation of glycosaminoglycans such as keratan sulfate and chondroitin-6-sulfate. GALNS is a lysosomal sulfatase required for the stepwise degradation of these sulfated glycosaminoglycans; its deficiency causes mucopolysaccharidosis IVA, a condition characterized by pathological GAG accumulation and ECM disruption.^15^^–^^18^ Under normal physiological conditions, GALNS may ensure proper turnover of ECM components, preventing pathological accumulation that could compromise tissue architecture. However, in the context of advanced PDR with tractional complications, the baseline reduction of GALNS in patients with VH + TRD may reflect a maladaptive shift toward ECM dysregulation. This progressive enzyme deficiency could lead to abnormal accumulation of sulfated glycosaminoglycans within fibrovascular membranes, promoting structural rigidity and contractile dysfunction.^15^^–^^18^ The further decrease following anti-VEGF treatment suggests inadequate restoration of ECM homeostasis, potentially exacerbating matrix dysfunction. Notably, independent PRM validation in an expanded cohort that included non-diabetic cataract controls revealed a biphasic pattern: GALNS was significantly elevated in PDR aqueous humor relative to cataract controls, yet progressively depleted in VH + TRD compared with simple VH and further declined after anti-VEGF treatment. One possible explanation is that vitreous hemorrhage initially leads to leakage of blood-derived GALNS into the aqueous humor, which may exert a transient protective anti-fibrotic effect that diminishes as GALNS is progressively consumed in more severe disease stages; however, this hypothesis remains speculative, as no paired blood and aqueous humor GALNS measurements were performed. Alternatively, GALNS elevation may reflect a compensatory upregulation by local ocular tissues in response to PDR-related pathological stimuli, with subsequent failure of this compensatory mechanism in advanced disease and further suppression following anti-VEGF treatment. Future studies incorporating paired blood–aqueous humor GALNS measurements and serial post-injection sampling would help distinguish between these possibilities. However, GALNS was thus confirmed as the only aggravation-type molecule validated on both cross-sectional and longitudinal comparison axes. Furthermore, siRNA-mediated GALNS knockdown in fibroblasts directly promoted myofibroblast activation—evidenced by upregulation of α-SMA and collagen I—accelerated cell migration, and enhanced gel contractility, recapitulating the three cardinal features of crunch syndrome. These convergent lines of evidence—discovery-phase omics, independent targeted validation, and in vitro functional experiments—position GALNS as a central mediator in the pro-fibrotic cascade triggered by anti-VEGF therapy, and as the most promising translational candidate for perioperative risk stratification and adjunctive therapeutic targeting.^19^

The other two aggravation-type molecules offer additional, although less fully validated, mechanistic insights. CAST, the sole endogenous inhibitor of calpains,^20^^,^^21^ was elevated at baseline in patients with VH + TRD and further increased following anti-VEGF treatment. In the context of advanced PDR, sustained CAST upregulation may reflect a maladaptive response where excessive calpain inhibition disrupts the proteolytic processes necessary for ECM remodeling and fibrovascular membrane resolution.^22^^,^^23^ Similarly, 3-HPA acid, a key intermediate in cellular energy metabolism,^24^^,^^25^ was progressively reduced in patients with VH + TRD and further declined post-treatment, suggesting disrupted energy homeostasis that may compromise cellular repair mechanisms and contribute to the pro-fibrotic microenvironment. However, neither molecule was confirmed in the independent validation cohort, as within-PDR subgroup and longitudinal comparisons, all failed to reach statistical significance, likely reflecting limited statistical power. The mechanistic roles of these two molecules therefore remain hypothetical and warrant investigation in adequately powered cohorts.

Conversely, the broad spectrum of improvement-type molecules underscores the systemic benefits of anti-VEGF treatment beyond simple vascular inhibition. At the protein level, the normalization of ALB levels reflects the restoration of the blood-retinal barrier, a primary goal of therapy.^26^ The favorable modulation of ABHD12B and NIBAN3 suggests that anti-VEGF treatment also positively influences lipid metabolism and apoptosis pathways, contributing to overall retinal health.^27^ Metabolomically, the coordinated rebalancing of 32 metabolites involved in critical pathways such as amino acid (e.g., L-glutamic acid), purine (e.g., inosine), and fatty acid metabolism points to a comprehensive restoration of cellular homeostasis.^28^^–^^30^ This integrated response indicates that effective anti-VEGF therapy initiates a beneficial “vascular inhibition-metabolic optimization” cycle, where reduced vascular leakage and hypoxia allow for the normalization of the broader retinal microenvironment.

Our study’s design and multi-omics approach represent a significant advance over previous investigations. Many prior studies were limited by cross-sectional designs or a single-omic modality, which could not distinguish baseline disease severity from true therapeutic response^7^^,^^31^ Recent aqueous humor proteomic studies have characterized stage-dependent molecular alterations using data-independent acquisition with PRM validation,^6^ paired proteomic profiling of aqueous humor has compared different anti-VEGF agents,^32^ and proximity extension assay proteomics in 121 patients with PDR has delineated novel molecular endotypes.^33^ Machine learning-assisted multi-omic analyses—encompassing aqueous humor metabolomics and lipidomics in a cohort of 108 patients,^34^ aqueous humor proteomics,^35^ and dual vitreous-plasma metabolic fingerprinting^36^—have further yielded predictive signatures for DR classification and anti-VEGF outcome prediction. Whether or not incorporating machine learning, these investigations—like prior transcriptomic^31^ and single-omic^37^^,^^38^ studies—were constrained by cross-sectional designs, single-omic coverage, or the absence of stratification by baseline tractional retinal detachment status. By combining baseline stratification with a longitudinal paired design, our study is one of the first to systematically differentiate these variables, linking a patient’s initial molecular state to their subsequent therapeutic trajectory and providing a more robust interpretation of the molecular cascade. This integrative strategy aligns with the emerging paradigm of using liquid-biopsy multi-omics to deconstruct complex ocular pathologies at a cellular level.^11^

Several limitations should be acknowledged. First, although our discovery cohort (*n* = 25 paired samples) provided adequate statistical power for identifying differentially expressed molecules, the validation subgroups were limited in size (control, *N* = 10; simple VH, *n* = 5; and VH + TRD, *n* = 5). This likely explains why CAST and 3-HPA acid, despite showing consistent trends in the discovery phase, did not reach statistical significance in the validation cohort. Accordingly, GALNS is the sole robustly validated aggravation-type molecule, whereas CAST and 3-HPA acid should be regarded as promising candidates warranting future validation in larger, multicenter cohorts. Second, our 7-day post-injection sampling time point captures the early molecular response window but may not reflect the peak fibrotic phase, as crunch syndrome typically manifests 1 to 6 weeks after anti-VEGF injection. Importantly, we do not have long-term clinical follow-up data to determine whether early GALNS depletion predicts subsequent crunch syndrome development. The current findings therefore establish a molecular association rather than a predictive relationship. A prospective study with serial aqueous humor sampling and clinical outcome tracking at 2 to 6 weeks post-injection would be necessary to establish the prognostic value of early GALNS changes. Third, our in vitro functional validation was performed using L929 murine fibroblasts rather than primary human retinal fibroblasts or Müller cells, which limits the direct translational relevance. Although L929 cells are a well-established model for fibroblast-to-myofibroblast transition, confirmation in primary human ocular fibroblasts or patient-derived cells would strengthen the clinical applicability of GALNS. Fourth, our proposed mechanism of blood-derived GALNS leakage through vitreous hemorrhage remains speculative and requires direct experimental verification, as discussed above. Finally, as a hypothesis-driven mechanistic study, machine learning was not used; but GALNS represents a priority candidate for future machine learning-based risk stratification models.

## Conclusions

This study provides integrated proteo-metabolomic evidence explaining the “double-edged sword” effect of preoperative anti-VEGF therapy in PDR. By identifying distinct molecular signatures of both therapeutic improvement and fibrotic aggravation—with GALNS independently validated and functionally confirmed as a key pro-fibrotic mediator—we offer a mechanistic foundation for the crunch syndrome and provide a critical scientific basis for developing personalized perioperative strategies to mitigate this risk.

## Supplementary Material

Supplement 1

Supplement 2

Supplement 3

Supplement 4

Supplement 5

Supplement 6

Supplement 7

Supplement 8

Supplement 9

Supplement 10

Supplement 11
