# Supplementary figures and images for: Longitudinal Multi-Omics Profiling of Aqueous Humor Implicates GALNS Depletion as a Pro-Fibrotic Mediator of Anti-VEGF Therapy in PDR
Source: Invest Ophthalmol Vis Sci. 2026 May 18;67(5):43. doi: 10.1167/iovs.67.5.43 (PMC13193207; doi:10.1167/iovs.67.5.43)

Figure 5: Improvement Molecules Expression Patterns

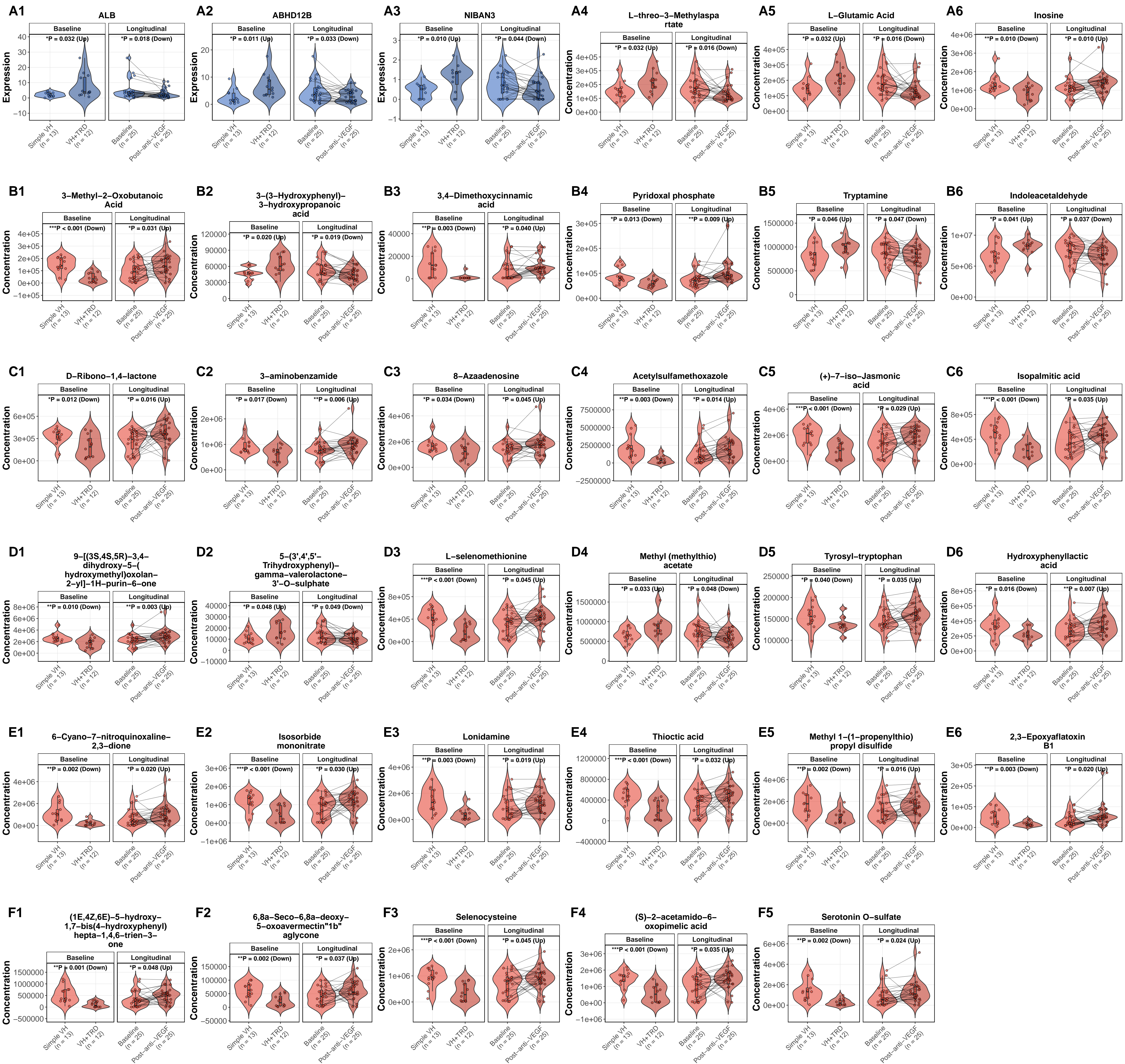

Supplement: Supplement 1 [file iovs-67-5-43_s001.pdf]

A

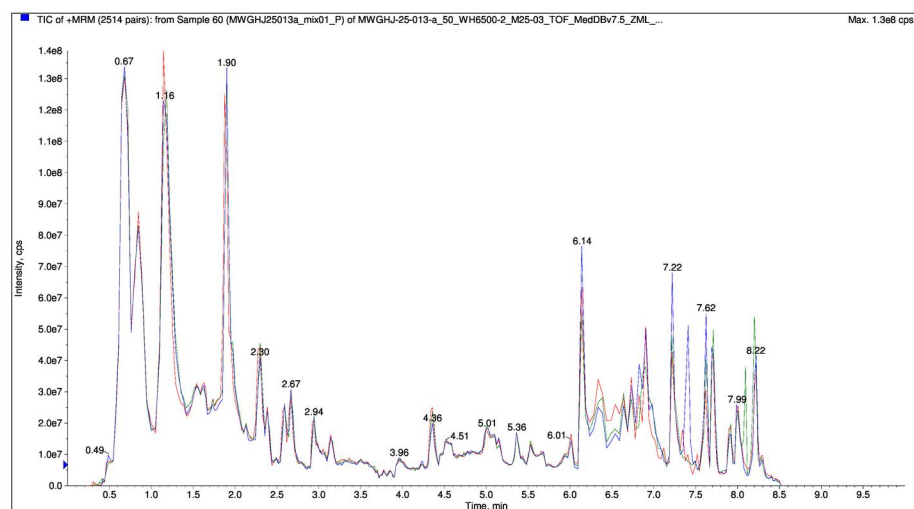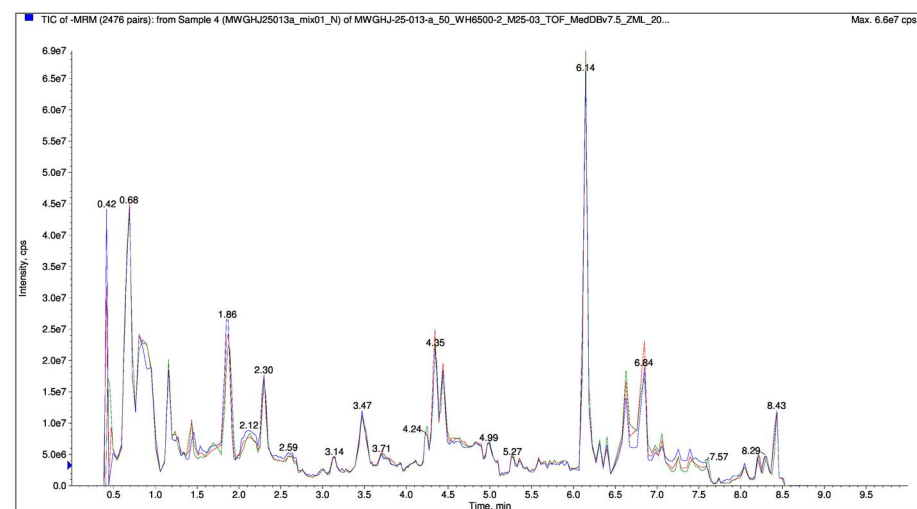

ESI+

ESI-

B

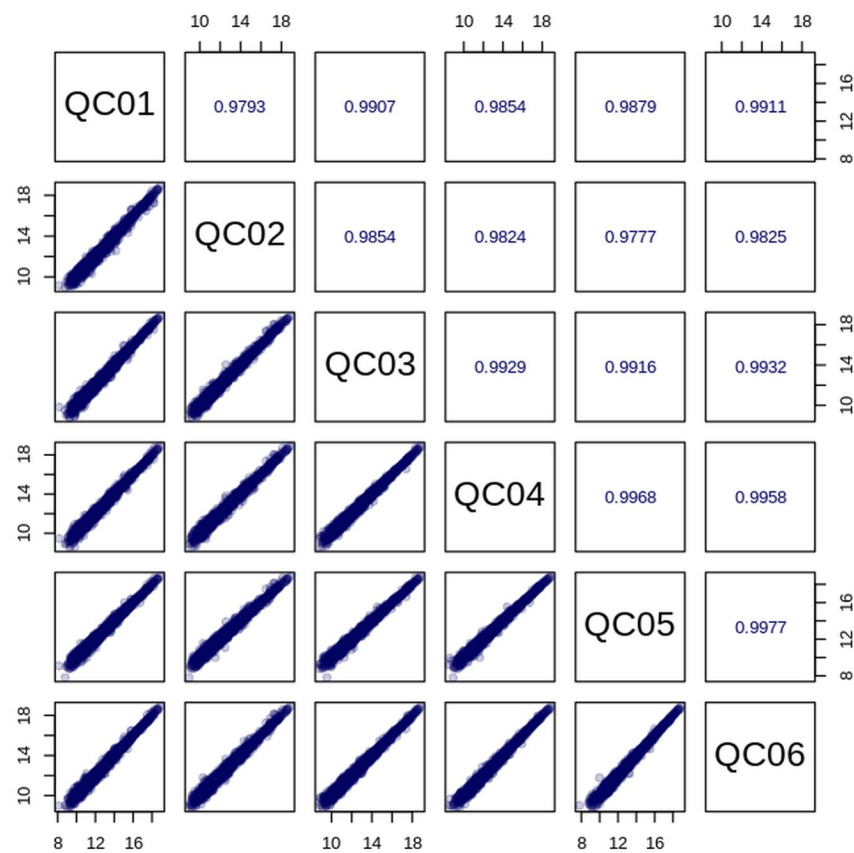

C

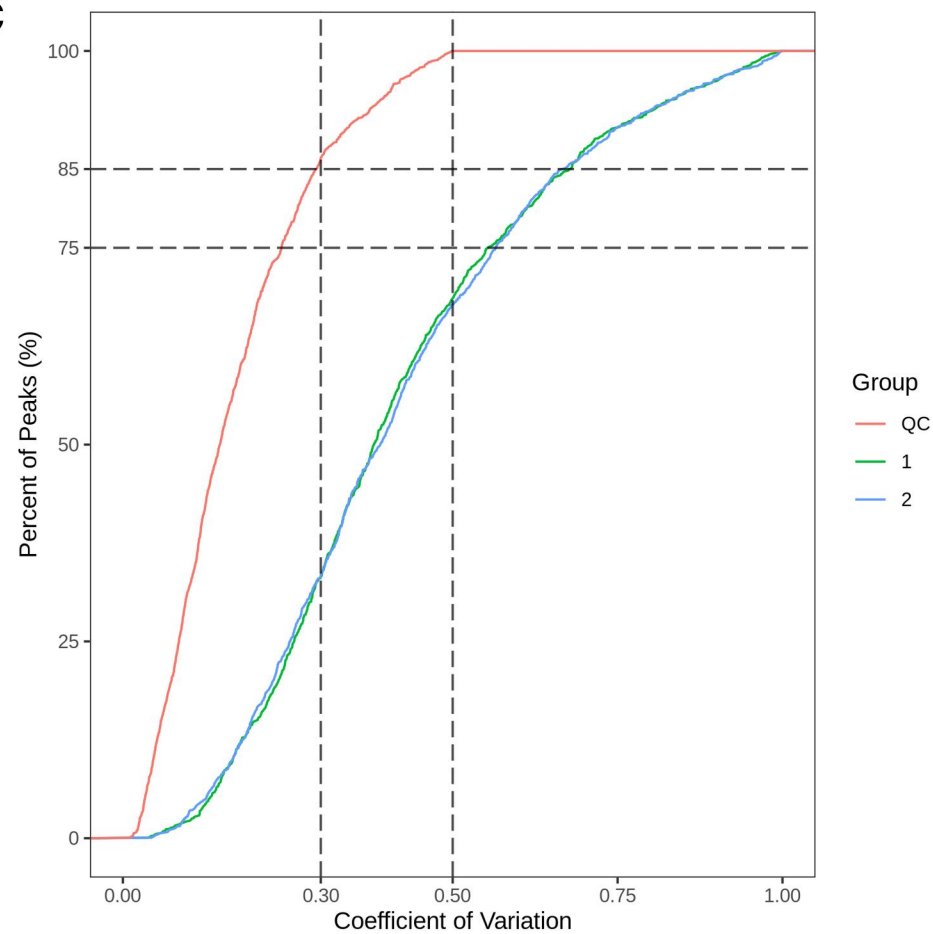

Supplement: Supplement 3 [file iovs-67-5-43_s003.pdf]

**A****PRM Peptide Detection Rate (n = 30 runs)**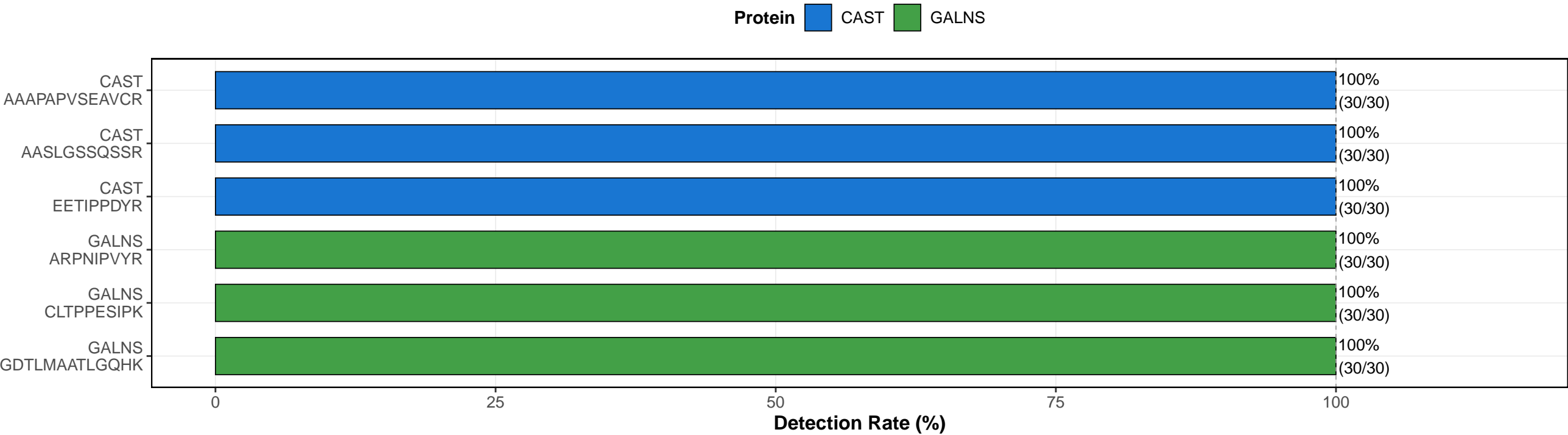**B****iRT Internal Standard Intensity Stability (n = 30 runs)**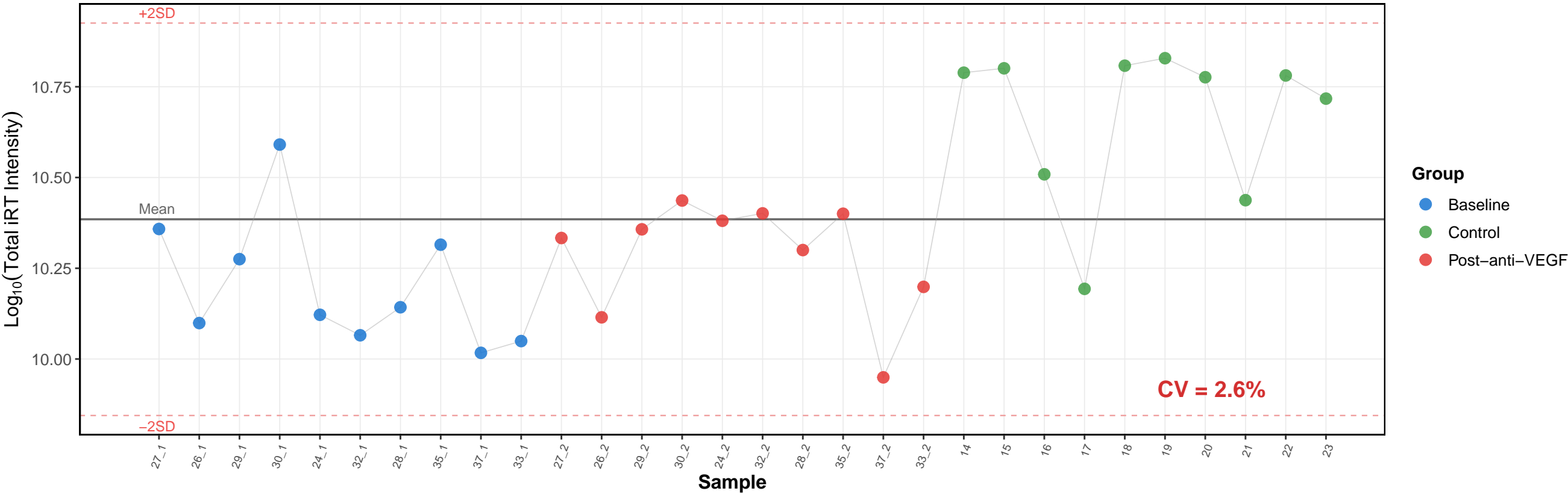

Supplement: Supplement 4 [file iovs-67-5-43_s004.pdf]

**A****3-HPA QC Sample Precision (n = 4)**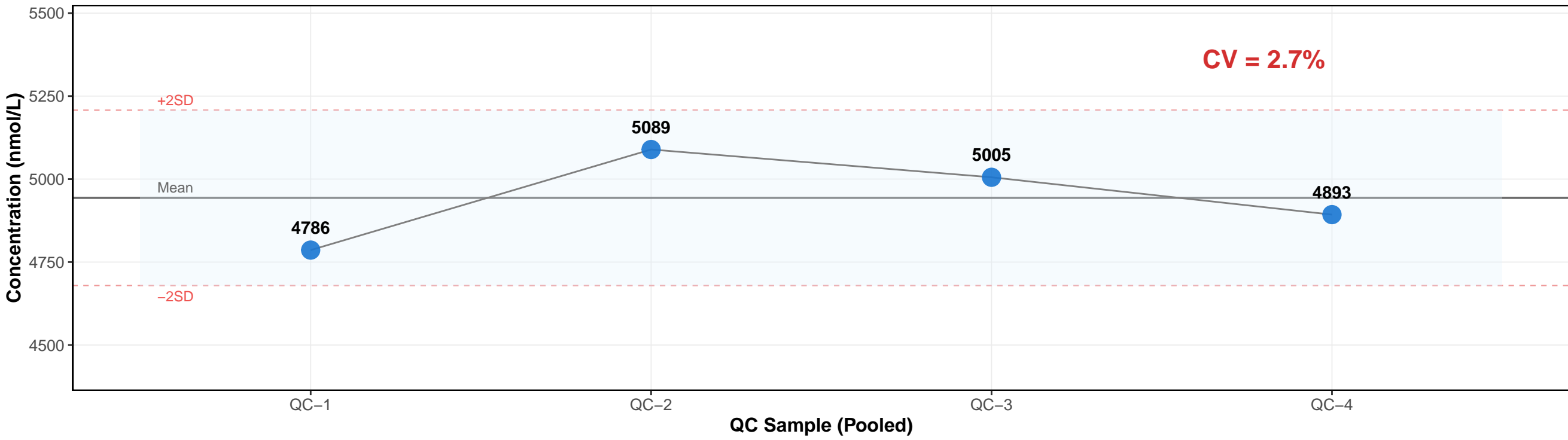**B****3-HPA-D4 Internal Standard Stability (n = 34 runs)**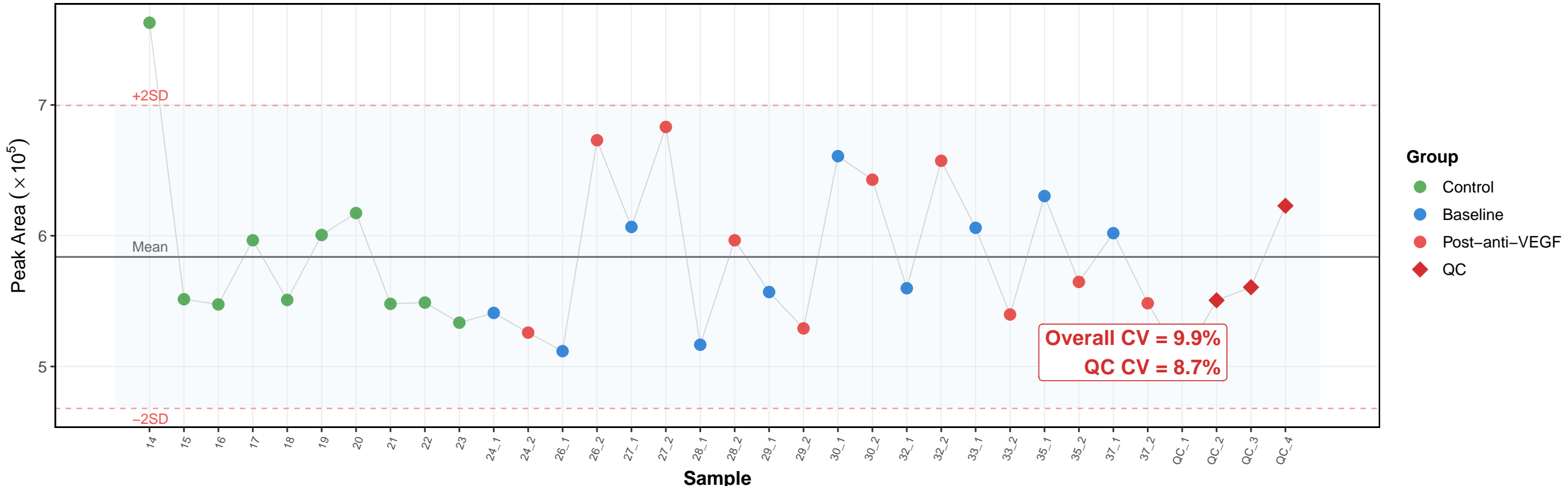

Supplement: Supplement 5 [file iovs-67-5-43_s005.pdf]
