# Supplementary material for: Longitudinal Multi-Omics Profiling of Aqueous Humor Implicates GALNS Depletion as a Pro-Fibrotic Mediator of Anti-VEGF Therapy in PDR
Source: Invest Ophthalmol Vis Sci. 2026 May 18;67(5):43. doi: 10.1167/iovs.67.5.43 (PMC13193207; doi:10.1167/iovs.67.5.43)
Supplement: Supplement 2 [file iovs-67-5-43_s002.pdf]

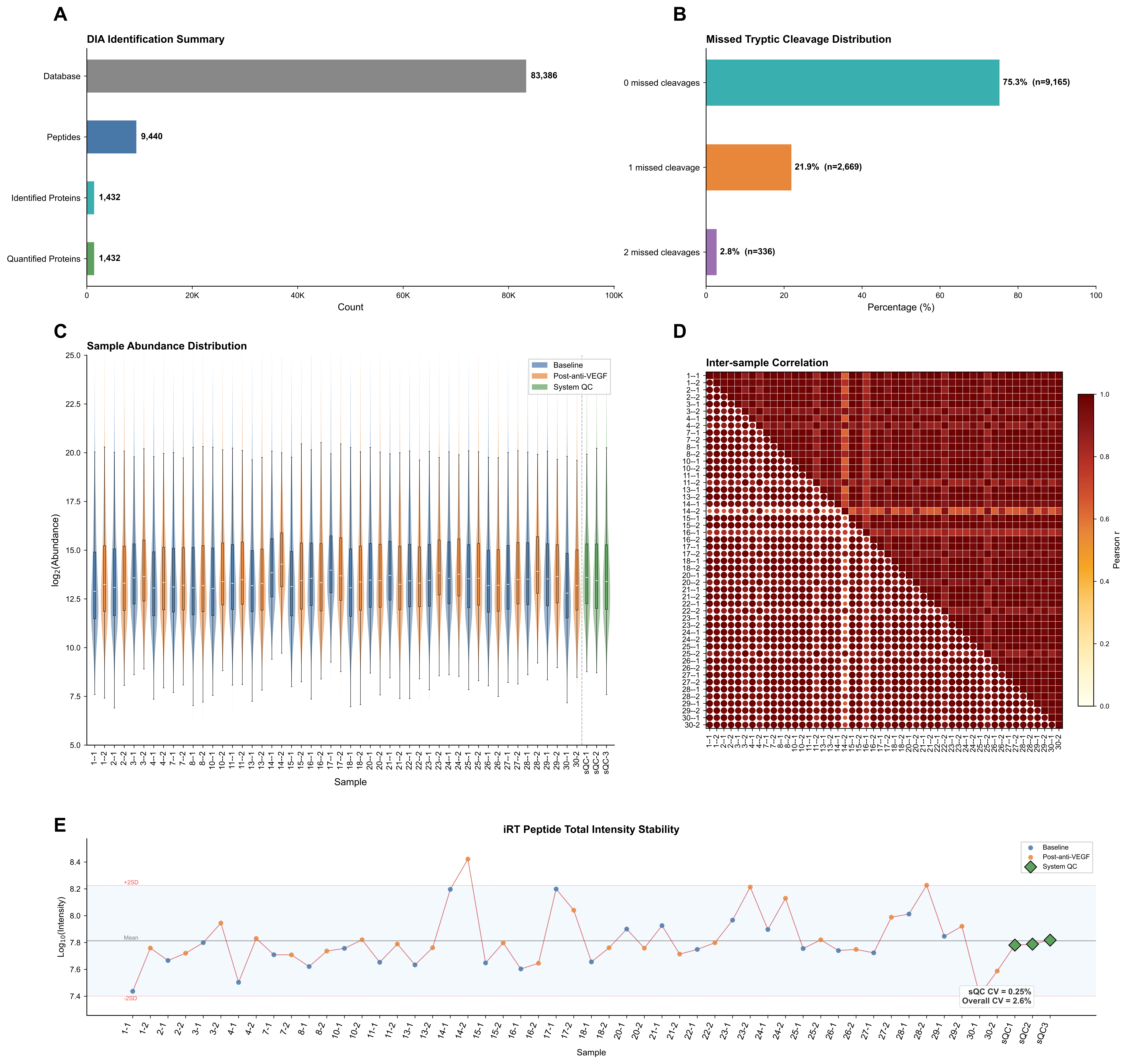
A
DIA Identification Summary
Database
83,386
Peptides
9,440
Identified Proteins
1,432
Quantified Proteins
1,432
0
20K
40K
60K
80K
100K
Count
B
Missed Tryptic Cleavage Distribution
0 missed cleavages
75.3% (n=9,165)
1 missed cleavage
21.9% (n=2,669)
2 missed cleavages
2.8% (n=336)
0
20
40
60
80
100
Percentage (%)
C
Sample Abundance Distribution
Baseline
Post-anti-VEGF
System QC
log2(Abundance)
Sample
D
Inter-sample Correlation
Pearson r
E
iRT Peptide Total Intensity Stability
+2SD
Mean
-2SD
Log10(Intensity)
Baseline
Post-anti-VEGF
System QC
sQC CV = 0.25%
Overall CV = 2.6%
Sample
