# Supplementary material for: Longitudinal Multi-Omics Profiling of Aqueous Humor Implicates GALNS Depletion as a Pro-Fibrotic Mediator of Anti-VEGF Therapy in PDR
Source: Invest Ophthalmol Vis Sci. 2026 May 18;67(5):43. doi: 10.1167/iovs.67.5.43 (PMC13193207; doi:10.1167/iovs.67.5.43)
Supplement: Supplement 6 [file iovs-67-5-43_s006.pdf]

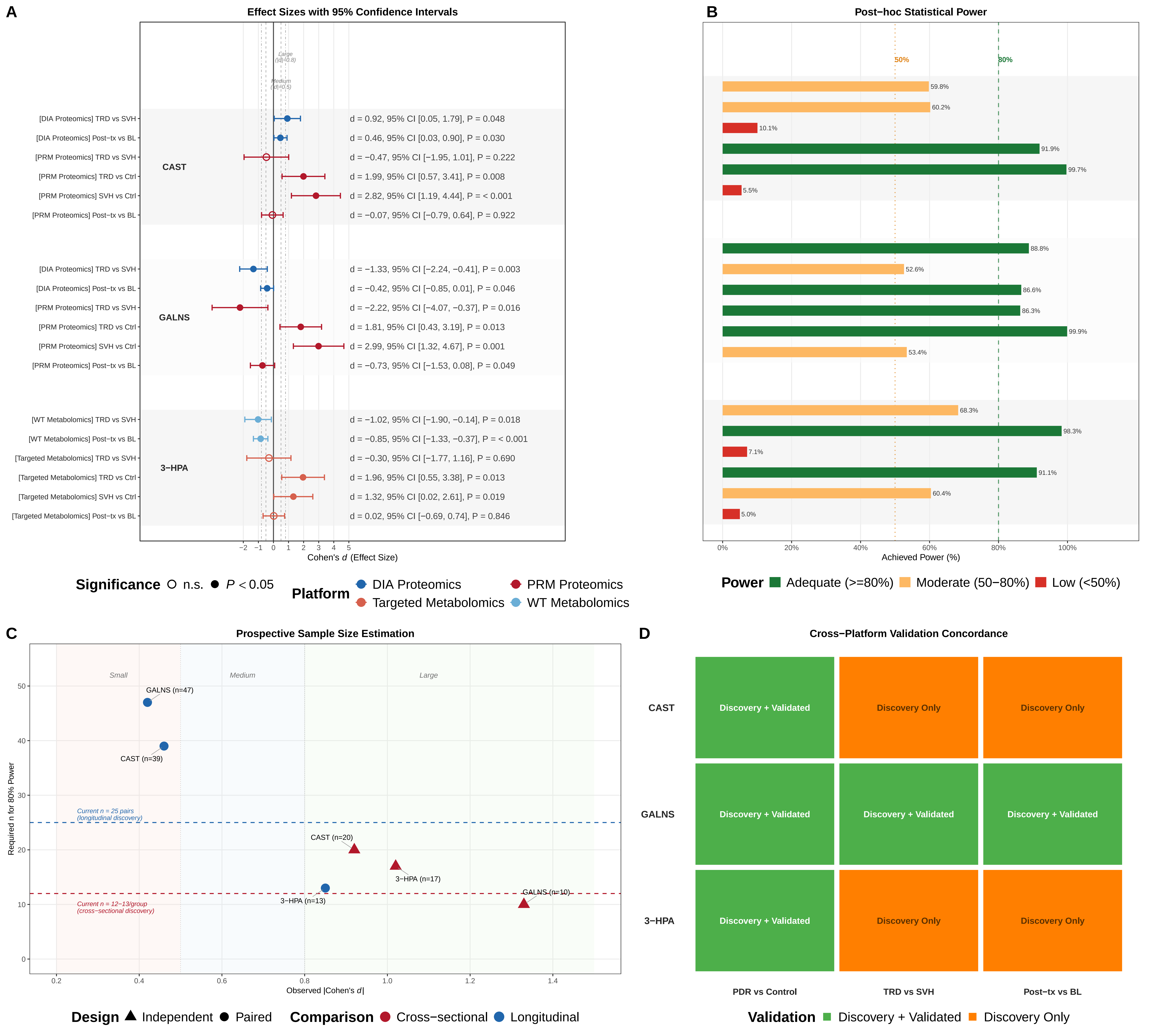
A
Effect Sizes with 95% Confidence Intervals
CAST
[DIA Proteomics] TRD vs SVH
d = 0.92, 95% CI [0.05, 1.79], P = 0.048
[DIA Proteomics] Post−tx vs BL
d = 0.46, 95% CI [0.03, 0.90], P = 0.030
[PRM Proteomics] TRD vs SVH
d = −0.47, 95% CI [−1.95, 1.01], P = 0.222
[PRM Proteomics] TRD vs Ctrl
d = 1.99, 95% CI [0.57, 3.41], P = 0.008
[PRM Proteomics] SVH vs Ctrl
d = 2.82, 95% CI [1.19, 4.44], P = < 0.001
[PRM Proteomics] Post−tx vs BL
d = −0.07, 95% CI [−0.79, 0.64], P = 0.922
GALNS
[DIA Proteomics] TRD vs SVH
d = −1.33, 95% CI [−2.24, −0.41], P = 0.003
[DIA Proteomics] Post−tx vs BL
d = −0.42, 95% CI [−0.85, 0.01], P = 0.046
[PRM Proteomics] TRD vs SVH
d = −2.22, 95% CI [−4.07, −0.37], P = 0.016
[PRM Proteomics] TRD vs Ctrl
d = 1.81, 95% CI [0.43, 3.19], P = 0.013
[PRM Proteomics] SVH vs Ctrl
d = 2.99, 95% CI [1.32, 4.67], P = 0.001
[PRM Proteomics] Post−tx vs BL
d = −0.73, 95% CI [−1.53, 0.08], P = 0.049
3−HPA
[WT Metabolomics] TRD vs SVH
d = −1.02, 95% CI [−1.90, −0.14], P = 0.018
[WT Metabolomics] Post−tx vs BL
d = −0.85, 95% CI [−1.33, −0.37], P = < 0.001
[Targeted Metabolomics] TRD vs SVH
d = −0.30, 95% CI [−1.77, 1.16], P = 0.690
[Targeted Metabolomics] TRD vs Ctrl
d = 1.96, 95% CI [0.55, 3.38], P = 0.013
[Targeted Metabolomics] SVH vs Ctrl
d = 1.32, 95% CI [0.02, 2.61], P = 0.019
[Targeted Metabolomics] Post−tx vs BL
d = 0.02, 95% CI [−0.69, 0.74], P = 0.846
Cohen's d (Effect Size)
Significance n.s. P < 0.05
Platform DIA Proteomics PRM Proteomics Targeted Metabolomics WT Metabolomics
B
Post−hoc Statistical Power
50%
80%
59.8%
60.2%
10.1%
91.9%
99.7%
5.5%
88.8%
52.6%
86.6%
86.3%
99.9%
53.4%
68.3%
98.3%
7.1%
91.1%
60.4%
5.0%
0% 20% 40% 60% 80% 100%
Achieved Power (%)
Power Adequate (>=80%) Moderate (50−80%) Low (<50%)
C
Prospective Sample Size Estimation
Small
Medium
Large
GALNS (n=47)
CAST (n=39)
Current n = 25 pairs
(longitudinal discovery)
CAST (n=20)
3−HPA (n=17)
3−HPA (n=13)
GALNS (n=10)
Current n = 12−13/group
(cross−sectional discovery)
Required n for 80% Power
Observed |Cohen's d|
Design Independent Paired
Comparison Cross−sectional Longitudinal
D
Cross−Platform Validation Concordance
CAST
Discovery + Validated
Discovery Only
Discovery Only
GALNS
Discovery + Validated
Discovery + Validated
Discovery + Validated
3−HPA
Discovery + Validated
Discovery Only
Discovery Only
PDR vs Control
TRD vs SVH
Post−tx vs BL
Validation Discovery + Validated Discovery Only
