# Supplementary material for: Longitudinal Multi-Omics Profiling of Aqueous Humor Implicates GALNS Depletion as a Pro-Fibrotic Mediator of Anti-VEGF Therapy in PDR
Source: Invest Ophthalmol Vis Sci. 2026 May 18;67(5):43. doi: 10.1167/iovs.67.5.43 (PMC13193207; doi:10.1167/iovs.67.5.43)
Supplement: Supplement 7 [file iovs-67-5-43_s007.pdf]

Supplementary Methods

2.3 DIA-Based Proteomics Workflow

Aqueous humor samples were thawed on ice from −80°C, and phenylmethylsulfonyl fluoride (PMSF) was added to a final concentration of 1 mM. Samples were centrifuged at 4,500 × g for 10 min at 4°C, and the resulting supernatant was collected. Protein concentration was determined using a BCA protein assay kit (Beyotime, Shanghai, China) and, where necessary, concentrated using 10 kDa molecular weight cut-off ultrafiltration devices (Beckman Coulter Inc., Brea, CA). Protein extraction quality control confirmed that all samples passed quality assessment and were included in downstream analysis.

For enzymatic digestion, a 100 μg aliquot of protein from each sample was adjusted to a volume of 200 μL with 8 M urea. Proteins were reduced with 5 mM dithiothreitol (DTT) at 37°C for 45 min and subsequently alkylated with 11 mM iodoacetamide (IAA) in the dark at room temperature for 15 min. The solution was diluted with 800 μL of 25 mM ammonium bicarbonate, followed by overnight digestion with trypsin (Promega, V5280) at 37°C. Digestion was terminated by adjusting the pH to 2–3 with 20% trifluoroacetic acid (TFA). Peptides were desalted using C18 solid-phase extraction cartridges (Millipore, Billerica, MA), and the final peptide concentration was quantified using the Pierce Quantitative Peptide Assay (Thermo Fisher Scientific). Digestion efficiency was verified by the distribution of missed tryptic cleavage sites: 75.3% of identified peptides had zero missed cleavages, 21.9% had one, and 2.8% had two missed cleavages (Supplementary Figure S2).

Peptides (200 ng) were analyzed using a Vanquish Neo UHPLC system coupled to an Orbitrap Astral mass spectrometer (Thermo Fisher Scientific). Separation was performed on an Easy-Spray PepMap Neo UHPLC column (150 µm × 15 cm, 2 µm) preceded by a PepMap Neo Trap Cartridge (300 µm × 5 mm, 5 µm) at 55°C. A 6.9-minute effective gradient was employed at a flow rate of 2.5 µL/min, using 0.1% formic acid in water (mobile phase A) and 0.1% formic acid in acetonitrile (mobile phase B). Mass spectrometry was operated in data-independent acquisition (DIA) positive ion mode. Full MS scans were acquired over a range of 380–980 m/z at a resolution of 240,000 (at 200 m/z) with a Normalized AGC Target of 500% and Maximum IT of 5 ms. MS/MS spectra were acquired across 299 isolation windows (2 Th width) with HCD collision energy set to 25%, Normalized AGC Target of 500%, and Maximum IT of 3 ms. All samples were analyzed within a single LC-MS/MS analytical batch with randomized injection order to minimize systematic bias from instrument drift.

Protein identification and quantification were performed using DIA-NN (v1.8.1) in library-free mode against the UniProt human proteome database (UP000005640; 83,386 entries; downloaded February 24, 2025), utilizing deep learning-based spectral library prediction and the match-between-runs (MBR) feature. The false discovery rate (FDR) was controlled at < 1% at both precursor and protein levels, and protein quantification was based on the MaxLFQ algorithm. A total of 9,440 unique peptides and 1,432 protein groups were identified and quantified, achieving a 100% quantification rate.

For both proteomic and metabolomic datasets, data were processed independently. Features present in over 50% of samples within at least one group were retained; features below this threshold were removed to eliminate noise from sporadic, low-confidence identifications. Missing values were imputed using the

K-nearest neighbors (KNN) algorithm. Protein abundance data were log2-transformed and normalized by the median, where the relative abundance of each protein was calculated as the ratio of its raw intensity to the median intensity of the corresponding sample. Metabolite data underwent log2 transformation and were normalized using a quantile-based method. Outlier samples were identified and excluded based on pre-established criteria, including total ion current intensity, number of identified features, or a Z-score > 3.

All samples were processed in a single sample preparation batch and analyzed in a single LC-MS/MS analytical batch to minimize technical variability. Sample injection order was randomized prior to acquisition. Pooled quality control (QC) samples, prepared by mixing equal aliquots from all samples, were injected at regular intervals throughout the analytical sequence to monitor instrument performance. Instrument stability was further monitored by the indexed retention time (iRT) peptide standard spiked into each sample; the total intensity of iRT peptides showed a coefficient of variation (CV) of 0.25% for system QC injections and an overall CV of 2.6% across all 53 runs. Potential batch effects were further assessed by inter-sample Pearson correlation analysis and evaluation of sample abundance distributions. Correlation coefficients were uniformly high with no batch-related substructure, and abundance distributions showed consistent median intensities across all runs. These assessments confirmed the absence of significant batch effects, and no post-hoc batch correction was required.

2.4 Widely-Targeted Metabolomics

Widely-targeted metabolomic profiling of aqueous humor samples was performed using an integrated UPLC-MS/MS widely-targeted metabolomics approach for large-scale metabolite detection, identification, and semi-quantification, as

previously described[1] This workflow has been adopted in recent high-impact metabolomics studies using the same analytical platform.[2,3]

Aqueous humor samples (50 μL) were thawed on ice, and 150 μL of extraction solvent (acetonitrile:methanol, 1:4, v/v) containing isotope-labeled internal standards was added. The mixture was vortexed for 3 min, centrifuged at 12,000 rpm for 10 min at 4°C, and incubated at −20°C for 30 min. After a second centrifugation, 120 μL of supernatant was transferred for LC-MS/MS analysis. Internal standards spanning multiple chemical classes were spiked into each sample to monitor extraction efficiency and instrument stability, including isotope-labeled compounds (FFA(16:0)-d31, L-leucine-d7, Citric acid-d4, L-Tryptophan-d5, Hexanoic acid-d11, benzoic acid-d5, and Caffeine-trimethyl-^{13}C3) and a non-endogenous synthetic standard (L-2-chlorophenylalanine). All internal standards demonstrated excellent reproducibility with coefficients of variation (CV) ranging from 2.1% to 5.6% across all injections.

An aliquot from each sample was pooled to create a quality control (QC) sample, which was first subjected to full-scan acquisition on a TripleTOF 6600 system (AB SCIEX) in information-dependent acquisition (IDA) mode using Analyst TF 1.7.1 software for metabolite annotation against the MetWare in-house metabolite database (MedDB v7.5) containing reference standards. The annotated ion pair information (precursor ion Q1, product ion Q3, and retention time) was then transferred to a QTRAP® 6500+ system (SCIEX) for scheduled multiple reaction monitoring (MRM) analysis of individual samples. A total of 2,514 MRM ion pairs were monitored across positive and negative ESI modes.

Chromatographic separation was performed on an ACQUITY UPLC HSS T3 C18 column (1.8 μm, 2.1 mm × 100 mm, Waters) using an ExionLC AD system (SCIEX)

at a flow rate of 0.4 mL/min and column temperature of 40°C, with an injection volume of 2 μL. For the T3 column (positive and negative ion modes), the mobile phase consisted of 0.1% formic acid in water (A) and 0.1% formic acid in acetonitrile (B), with the following gradient: 0–2 min, 5–20% B; 2–5 min, 20–60% B; 5–6 min, 60–99% B; 6–7.5 min, 99% B (hold); 7.5–7.6 min, 99–5% B; 7.6–10 min, 5% B (re-equilibration). For hydrophilic metabolites, a complementary separation was performed on an ACQUITY UPLC BEH HILIC column (1.7 μm, 1 mm × 150 mm, Waters) in negative ion mode.

The QTRAP 6500+ mass spectrometer was operated with the following parameters: ESI source temperature 500°C; ion spray voltage 5,500 V (positive) and −4,500 V (negative); ion source gas I (GSI) 50 psi; ion source gas II (GSII) 50 psi; curtain gas (CUR) 25 psi; collision-activated dissociation (CAD) gas set to high. Data acquisition and processing were controlled by Analyst 1.6.3 software (SCIEX). A specific set of MRM transitions was monitored for each chromatographic period according to the metabolites eluted within that period.

Pooled QC samples were injected at regular intervals throughout the analytical sequence to monitor instrument performance. Pearson correlation coefficients among six QC injections ranged from 0.977 to 0.998, and more than 85% of detected peaks exhibited CV values below 0.3, confirming excellent instrument stability and quantitative reproducibility (Supplementary Figure S3).

Metabolite identification confidence was classified according to a four-tier system aligned with the Metabolomics Standards Initiative (MSI) criteria[4]: Levels 1a and 1b (n = 901, 58.4%) were identified by matching Q1, Q3, retention time, and MS2 spectra against authenticated reference standards (corresponding to MSI Level 1); Levels 2 and 3 (n = 641, 41.6%) were putatively annotated based on spectral

similarity without available reference standards (corresponding to MSI Level 2–3). Relative quantification was based on MRM peak areas normalized to internal standards. All detected metabolites were cross-referenced against the Human Metabolome Database (HMDB) and Kyoto Encyclopedia of Genes and Genomes (KEGG) to confirm endogenous origin.

A total of 1,542 metabolites were quantified by the MRM-based workflow. Features present in >50% of samples within at least one group were retained for downstream statistical analysis.

2.5 PRM-Based Targeted Proteomics Validation

To independently validate the aggravation-type proteins CAST and GALNS identified from the discovery-phase DIA proteomics, parallel reaction monitoring (PRM) was performed by Biotree Biotech Co., Ltd. (Shanghai, China).

Aqueous humor samples were thawed, vortexed, and centrifuged at 12,000 rpm for 10 min at 4°C. Protein concentration was determined by BCA assay (Beyotime, Shanghai, China) using a 7-point BSA calibration curve (0.025–0.5 mg/mL, R^2 = 0.998). A 30 μg aliquot of protein was processed using an SP3 (Single-Pot Solid-Phase-enhanced Sample Preparation) kit (Biotree, Shanghai, China). Proteins were reduced and alkylated at 95°C for 20 min using the SP3 kit reagents, bound to SP3 magnetic beads, washed, and digested with trypsin at 37°C for 2.5 hours. Peptides were desalted using C18 cartridges and quantified by NanoDrop (A205; Thermo Fisher Scientific).

For PRM method development, target peptide sequences were imported into Skyline software, and DIA data from representative pooled samples were used to

screen suitable peptides for the inclusion list. Spectral libraries were generated using MSFragger and Prosit to optimize PRM acquisition parameters and scheduling windows.

Peptides (1 μg) were separated on a Vanquish Neo nano-UPLC system using an Easy-Spray HPLC column (150 μm ID × 15 cm; Thermo Fisher Scientific) with an 8-minute gradient at a flow rate optimized for nano-flow. Mobile phases were 0.1% formic acid in water (A) and 0.1% formic acid in 80% acetonitrile (B). Mass spectrometry data were acquired on an Q Exactive HF-X mass spectrometer (Thermo Fisher Scientific) in PRM mode with positive ion detection. The NSI source was operated at 2,100 V with an ion transfer tube temperature of 290°C. Full MS scans covered 250–1,000 m/z at a resolution of 240,000. MS2 scans covered 150–2,000 m/z with a normalized collision energy (NCE) of 25% and a maximum injection time of 200 ms. Data were analyzed using Skyline software (MacCoss Lab, University of Washington). All target peptides (3 proteotypic peptides each for CAST and GALNS) achieved a 100% detection rate across all 30 PRM runs, and instrument stability was confirmed by iRT peptide intensity monitoring (overall CV = 2.6%) (Supplementary Figure S4).

2.6 Targeted Metabolite Quantification

To independently validate the key aggravation-type metabolite 3-hydroxypropanoic acid (3-HPA) identified from the discovery-phase metabolomics, targeted absolute quantification was performed using UPLC-MS/MS with selected reaction monitoring (SRM) on the AccuQuanter platform (iPhenome Biotechnology Inc./Yunpukang, Dalian, China).

Aqueous humor samples (20 μL) were transferred to 1.5 mL EP tubes (Corning Axygen, USA), mixed with 80 μL of methanol:acetonitrile (1:1, v/v), vortexed at 1,500 rpm for 3 min, and centrifuged at 12,000 × g for 20 min. The supernatant (40 μL) was transferred to a new tube, spiked with 5 μL of isotope-labeled internal standard mixture (0.4–400 μg/mL), 20 μL of 200 mM 3-nitrophenylhydrazine (3-NPH), and 20 μL of 200 mM 1-ethyl-3-(3-dimethylaminopropyl)carbodiimide (EDC), then incubated at 30°C for 60 min for chemical derivatization. After vacuum drying (Labconco Corporation, USA), the residue was reconstituted in 800 μL of 50% methanol for LC-MS/MS analysis.

An isotope-labeled internal standard (3-hydroxypropanoic acid-D4 sodium salt; Toronto Research Chemicals, Canada) was used for calibration. Stock solutions were prepared at 1.0 mg/mL and serially diluted to generate multi-point calibration curves for absolute quantification (nmol/L).

LC-MS/MS analysis was performed on a Thermo Ultimate 3000 UPLC coupled to a TSQ Endura MD Plus triple quadrupole mass spectrometer (Thermo Fisher Scientific) with SRM acquisition. Chromatographic separation was achieved on an ACQUITY UPLC HSS C18 column (1.8 μm, 2.1 × 100 mm) at a flow rate of 0.3 mL/min, using 0.01% formic acid in water (A) and acetonitrile (B) as mobile phases. The gradient was: 0–16 min, 15–40% B; 16–23 min, 40–95% B; 23–26 min, 95% B (hold); 26–27 min, 95–15% B; 27–30 min, 15% B (re-equilibration). The heated ESI source was operated in negative ion mode at −3.0 kV with an ion source temperature of 350°C; sheath gas 40 Arb, auxiliary gas 15 Arb, and collision gas 2.0 mTorr. Data were processed using TraceFinder software (Thermo Fisher Scientific).

Four pooled QC samples were distributed evenly across the analytical sequence. For 3-hydroxypropanoic acid, quantification across the four pooled QC samples yielded a mean concentration of 4,943 nmol/L with a CV of 2.7%, and all measurements fell within ±2 standard deviations of the mean. The corresponding isotope-labeled internal standard (3-HPA-D4) exhibited stable peak areas across all 34 analytical runs (overall CV = 9.9%; QC CV = 8.7%), confirming consistent instrument performance throughout the batch (Supplementary Figure S5).

References

1. Chen W, Gong L, Guo Z, et al. A novel integrated method for large-scale detection, identification, and quantification of widely targeted metabolites: application in the study of rice metabolomics. Mol Plant. 2013;6(6):1769-1780. doi:10.1093/mp/sst080

2. Xu YX, Liu LD, Zhu JY, et al. Alistipes indistinctus-derived hippuric acid promotes intestinal urate excretion to alleviate hyperuricemia. Cell Host Microbe. 2024;32(3):366-381.e9. doi:10.1016/j.chom.2024.02.001

3. Huang Z, Liu Y, Wu Y, et al. Gut mucosal mycobiome profiling in Crohn's disease uncovers an AMP-mediated anti-inflammatory effect of Cladosporium sphaerospermum. Nat Metab. Published online January 7, 2026. doi:10.1038/s42255-025-01420-9

4. Sumner LW, Amberg A, Barrett D, et al. Proposed minimum reporting standards for chemical analysis Chemical Analysis Working Group (CAWG) Metabolomics Standards Initiative (MSI). Metabolomics. 2007;3(3):211-221. doi:10.1007/s11306-007-0082-2
